# A Systematic Approach to the Development of Cilostazol Nanosuspension by Liquid Antisolvent Precipitation (LASP) and Its Combination with Ultrasound

**DOI:** 10.3390/ijms222212406

**Published:** 2021-11-17

**Authors:** Emilia Jakubowska, Bartłomiej Milanowski, Janina Lulek

**Affiliations:** Chair and Department of Pharmaceutical Technology, Faculty of Pharmacy, Poznan University of Medical Sciences, 6 Grunwaldzka Street, 60-780 Poznan, Poland; farmstos@ump.edu.pl

**Keywords:** nanosuspension, nanocrystals, drug nanoparticles, antisolvent precipitation, sonoprecipitation, cilostazol

## Abstract

Nanosizing is an approach to improve the dissolution rate of poorly soluble drugs. The first aim of this work was to develop nanosuspension of cilostazol with liquid antisolvent precipitation (LASP) and its combination with ultrasound. Second, to systematically study the effect of bottom-up processing factors on precipitated particles’ size and identify the optimal settings for the best reduction. After solvent and stabilizer screening, in-depth process characterization and optimization was performed using Design of Experiments. The work discusses the influence of critical factors found with statistical analysis: feed concentration, stabilizer amount, stirring speed and ultrasound energy governed by time and amplitude. LASP alone only generated particle size of a few microns, but combination with ultrasound was successful in nanosizing (d10 = 0.06, d50 = 0.33, d90 = 1.45 µm). Micro- and nanosuspension’s stability, particle morphology and solid state were studied. Nanosuspension displayed higher apparent solubility than equilibrium and superior dissolution rate over coarse cilostazol and microsuspension. A bottom-up method of precipitation-sonication was demonstrated to be a successful approach to improve the dissolution characteristics of poorly soluble, BCS class II drug cilostazol by reducing its particle size below micron scale, while retaining nanosuspension stability and unchanged crystalline form.

## 1. Introduction

Cilostazol (CIL) is a phosphodiesterase III inhibitor used for the treatment of intermittent claudication. Owing to antiplatelet, vasodilatory and antiproliferative action, it has additionally demonstrated beneficial clinical outcomes in other cardiovascular diseases (e.g., prevention of secondary ischemic stroke) [1]. Recently, cilostazol has also been identified as a potential candidate for drug repurposing in the treatment of COVID-19 [2]. While its absolute bioavailability from tablets after oral administration in humans has not been determined, the drug is known to display a positive food effect [3], which may compromise the safety and efficacy of pharmacotherapy. Absorption increase after a high-fat meal is related to low aqueous solubility and slow dissolution rate in the gastrointestinal tract of this highly lipophilic, neutral compound of Biopharmaceutics Classification System (BCS) class II. 

Several approaches have been employed to overcome this and improve cilostazol dissolution, such as solid dispersions [4,5], inclusion complexes [6] or self-nanoemulsifying systems [7,8]. Among the pharmaceutical technologies applied to solve the problem of poor solubility and dissolution, an important place is held by drug nanocrystals or nanosuspensions. These are defined as active pharmaceutical ingredient (API) particles of average size under 1 µm, generally consisting of pure drug with a low amount of stabilizer excipients. Due to the reduction of particle size to submicron scale and the related increase in the solid’s surface area, the dissolution rate of such materials is increased in comparison to untreated or even micron-sized drug, according to the Noyes-Whitney equation (Equation (1)). Additionally, a certain increase in saturation solubility may also be expected, as nanoparticles exhibit higher curvature and this increases the dissolution pressure, according to the Ostwald-Freundlich equation (Equation (2)) [9,10,11].

(1) $$\frac{dC}{dt}=\frac{D\times A}{V\times h}\left(c_{s}-c_{t}\right)$$

**Equation (1).** Noyes-Whitney equation, where: *dC/dt*—dissolution rate; *D*—diffusion coefficient of a molecule; *A*—solid surface area; *V*—solvent volume; *h*—diffusion layer thickness; *c_s_*—saturation solubility; *c_t_*—solute concentration at the time *t*.

(2) $$S=S_{\infty }\exp\left(\frac{2\gamma M}{r\rho RT}\right)$$

**Equation (2).** Ostwald-Freundlich equation, where: *S*—saturation solubility of the nanosized API; *S_∞_*—saturation solubility of an infinitely large API crystal; *γ*—crystal-medium interfacial tension; *M*—API molecular weight; *r*—particle radius; *ρ*—density; *R*—gas constant; *T*—temperature.

The most popular, as well as industrially feasible, methods of nanocrystals production are top-down techniques, where large particles are mechanically fragmented into nanoparticles [12]. There are several examples of cilostazol nanosuspensions obtained with wet milling [13,14,15,16,17,18,19]. On the other hand, top-down methods are often relatively expensive and time- and energy consuming. Therefore, bottom-up techniques, where dissolved API is reprecipitated in a controlled manner to yield particles below 1 µm, are a simple, inexpensive alternative [20]. Among these, a popular approach to produce nanocrystals is liquid antisolvent precipitation (LASP), in which API dissolved in an appropriate solvent (usually organic) is precipitated upon addition of an antisolvent, where solubility is low (usually aqueous solution of polymeric and/or surfactant stabilizers) [21]. An in-depth explanation of this established process, the principles and the mechanism of supersaturation inducing the nucleation and their effect on the generated particle size can be found in excellent reviews on this topic [21,22]. LASP effectiveness can be further modified by the application of ultrasound during or immediately after the process, as this enhances crystal nucleation (by the mechanism of cavitation) and reduces their growth by deagglomeration, yielding particles of smaller size [23]. 

However, reports on using bottom-up methods with the aim to produce cilostazol nanosuspensions are rare. Kim et al. applied supercritical antisolvent precipitation for cilostazol dissolution enhancement, but the reported mean particle size is of a few microns [24]. Baek et al. reprecipitated cilostazol directly into an adsorbing carrier, which does not qualify as nanocrystals [25]. A typical LASP as a sole technique for cilostazol processing has been described by Sai Gouthami et al. [26] and Tari et al. [27]; however, these works aimed to modify cilostazol crystal habit instead of reducing the particles to nanosize, and consequently the reported particles are larger. 

On the other hand, Miao et al. used LASP from N-methyl-2-pyrrolidone with short intermittent sonication to obtain material in the low micron range, which was then subjected to high pressure homogenization in order to produce cilostazol nanosuspensions. In this case, the top-down processing step was essential in generating nanoparticles [28]. Finally, Choi has recently described the development of cilostazol nanocrystals via simultaneous sonoprecipitation or precipitation with homogenization. So far, this has been the only work to suggest that successful bottom-up generation of cilostazol nanoparticles is feasible [29]. However, it seems to focus mostly on reporting the final outcome of the designed material instead of attempts at systematic insight in the critical formulation and process parameters which affect the (sono)precipitated cilostazol particle size. Moreover, the particle sizes are presented for the material collected by centrifugation or filtration and the potential differences from the original nanosuspension sizes were not addressed in the work, similar to the unverified absence of larger particles since dynamic light scattering was the only method for the determination of particle size distribution. Interestingly, the effect of nanonization by LASP-sonication on the dissolution rate of cilostazol is not reported in Choi’s work, which makes it difficult to compare the results with the drug’s nanosuspensions developed with the use of other methods [29]. 

Given the scarcity of the existing reports on optimization of bottom-up methods in the context of developing cilostazol nanocrystals, a more in-depth investigation is warranted. The aim of this work is to fill the existing knowledge gaps by describing a systematic approach to the production of cilostazol nanosuspensions by liquid antisolvent precipitation and its combination with ultrasound. Step by step optimization of process and formulation parameters is described, including the solvent choice with the consideration of optimal supersaturation ratio [22] and stabilizer choice. Design of Experiment (DoE) is used to mathematically characterize the influence of LASP and sonication parameters on cilostazol particle size and to identify the optimal settings which result in the smallest nanoparticles. In this way, the relationships between bottom-up processing variables and nanosuspension characteristics are elucidated in a manner which so far has not been applied in the context of enhancing the dissolution of BCS class II drug cilostazol. 

## 2. Results and Discussion

**Figure 1:**
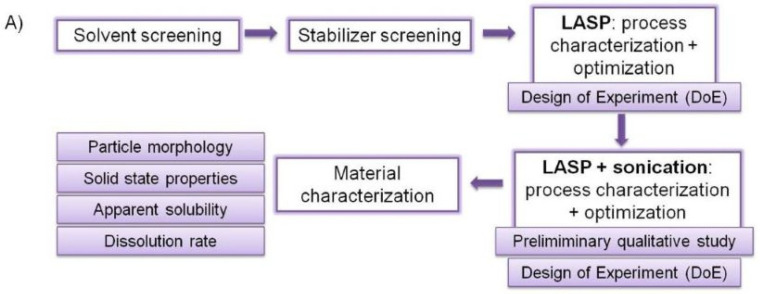

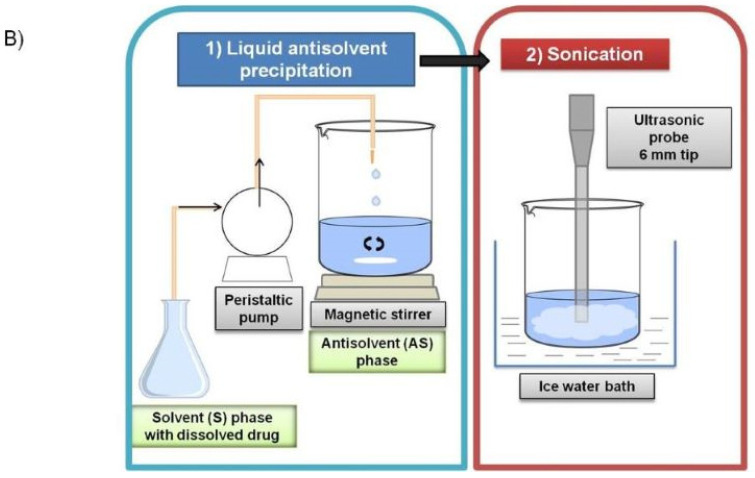
(**A**) A flowchart overview of the systematic approach in the current work. (**B**) A schematic diagram of liquid antisolvent precipitation and sonication process.

An overview of the workflow and consecutive stages of this systematic work is presented in Figure 1A. To avoid confounding the influence of qualitative formulation composition with quantitative processing parameters, which would not permit reliable scientific, statistical assessment of their effects on the precipitated particles sizes, the study started with the choice of most promising solvent. Next, stabilizers were screened to select the optimal polymer. Once the qualitative composition was established, in-depth characterization of the quantitative variables’ influence on cilostazol particle size was carried out according to DoE principles, with the aim to develop empirical mathematical models describing these relationships and pinpoint the optimal settings for the best particle size reduction. This was done separately for antisolvent precipitation itself and for its combination with ultrasound, for elucidation of the processes’ specific impact on the suspension size and as a logical consequence of the systematic approach to nanosuspension development and overcoming its challenges, as presented in detail in the following sections. Finally, the material produced in optimized LASP and LASP-sonication processes was subjected to physicochemical characterization. 

### 2.1. Solvent Screening

In the first step, screening studies for the most promising solvent phase were performed in two stages. Five organic solvents: acetic acid (AcOH), acetonitrile (ACN), dimethylformamide (DMF), dimethylsulfoxide (DMSO) and methanol (MeOH) were considered based on the solubilizing capacity for the drug [30], water miscibility, low toxicity (class 2 or 3 according to Ph.Eur.) and suitability for UV quantification of cilostazol. Additionally, PEG400 was considered as a more hydrophilic and environmentally friendly alternative. 

In principle, the precipitated particle size in LASP is governed by the competition of nucleation and crystal growth. High nucleation rates result in the formation of many small nuclei and supersaturation is the driving force of this phenomenon. As a simplified rule, high supersaturation ratio (SR), i.e., the ratio of the total compound concentration in the system to its equilibrium solubility, enhances the nucleation rate [20,21]. Therefore, in the first stage of solvent screening, the proportion of each solvent to water as antisolvent (S/AS ratio) was studied to identify the value where the highest supersaturation occurs (for details, see Appendix A). In the second stage of solvent screening, preliminary precipitation experiments were performed at S/AS values where highest SR was determined and additionally at SR values comparable between different solvents. To isolate the effect of solvent type and its S/AS, water without any stabilizers was chosen as antisolvent phase. The process is schematically represented in Figure 1B, and a detailed description is given in Section 3.2. Particle size distribution (PSD) of the precipitated suspension was immediately determined and the solvent-S/AS combination which yielded the lowest size was chosen for further studies.

**Figure A2:**
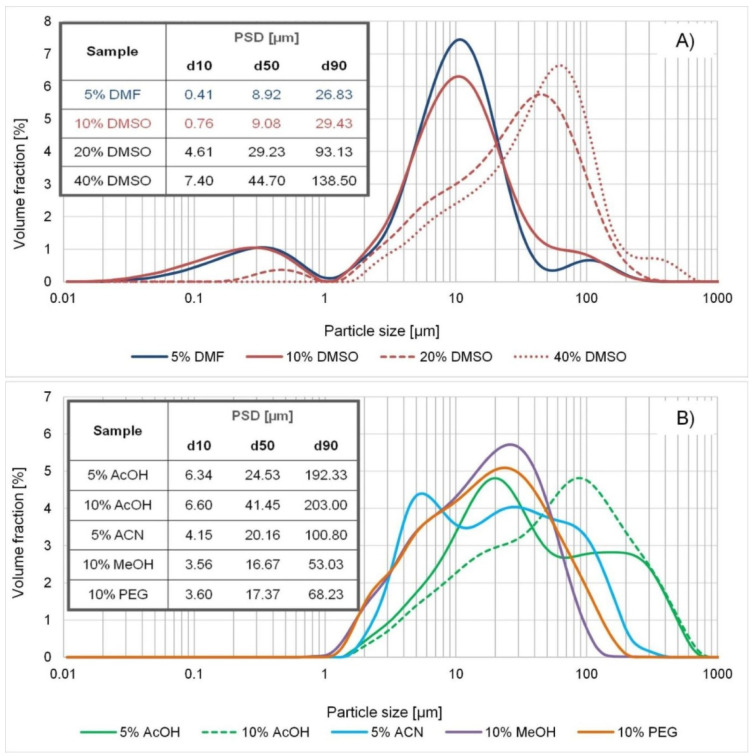
(**A**,**B**) Particle size distributions of cilostazol precipitated from different solvents without stabilizers at screening stage.

Unsurprisingly, none of the tested screening variants resulted in the precipitation of nanoparticles, since the conditions were not optimized, and stabilizers were absent. Based on the smallest PSD values two solvent-S/AS combinations were selected for further studies: 5% DMF and 10% DMSO, where the results were similar. The size distribution curves reflected three populations of particles, including a small fraction below 1 µm, which was not detected elsewhere (Figure A2, Appendix A). 

The results confirm that the precipitated particles’ size and presumed nucleation conditions are determined not only by the degree of supersaturation, but also by solvent characteristics (Table A1, Appendix A). An analysis of solvent properties revealed a qualitative correlation between CIL PSD and dielectric constant or log *p* value of the solvent. No clear correlation to properties such as solvent density, viscosity or surface tension was found. According to this, the most favorable solvents for cilostazol precipitation, DMSO and DMF, are the most hydrophilic ones of the tested range. This phenomenon most likely is explained by rapid micromixing conditions ensured by high affinity of polar solvents to water. The contact surface between S and AS phase is therefore relatively large and quickly developed, which results in immediate diffusion of API molecules to the interface and rapid nucleation to yield comparatively the smallest PSD [31]. Detailed literature discussion is presented in Appendix A. The LASP process of cilostazol from 10% DMSO or 5% DMF without stabilizers did not change its polymorphic form A [32] and did not generate solvates (data not shown), in agreement with the literature [26,27].

### 2.2. Stabilizer Screening

As the PSD of cilostazol precipitated from 10% DMSO and 5% DMF were similar, both solvents were considered in the stabilizer screening stage to choose the more advantageous combination. The processing parameters were kept the same as in Section 2.1 apart from the composition of AS phase. Different classes and molecular weights/grades of the most popular excipients used to stabilize nanosuspenions were considered, i.e., polymeric surface stabilizers: HPMC (E6, K4M, E50 type), PVA (9–10 kDa, 80% hydrolyzed and 30–70 kDa, 87–90% hydrolyzed), PVP K30, HPC; nonionic surfactants: PX188, PX407 and anionic surfactants: DOSS, SDS. The stabilizer concentration in water as antisolvent phase was set for screening purposes at 0.5% (*w*/*v*), except for SDS (0.002% to avoid exceeding critical micellar concentration and additional solubilization). 

Regardless of the stabilizer type, the resulting particle size distributions proved to be very close between the solvents, but in the case of 5% DMF there was a tendency for the growth of additional small population of ~100 µm (data not shown). Due to this and due to the potential of DMSO for higher feed concentrations, precipitation results from 10% DMSO were considered as the target ones and this solvent remained for further development stages.

**Figure 2:**
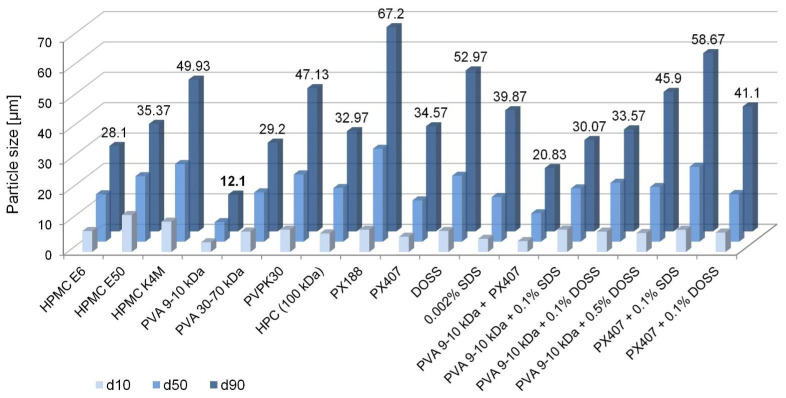
Particle size distribution values of cilostazol precipitated from 10% DMSO with different stabilizers during screening stage. Unless otherwise noted, stabilizer concentration was 0.5% *w*/*v* in antisolvent phase.

**Figure A3:**
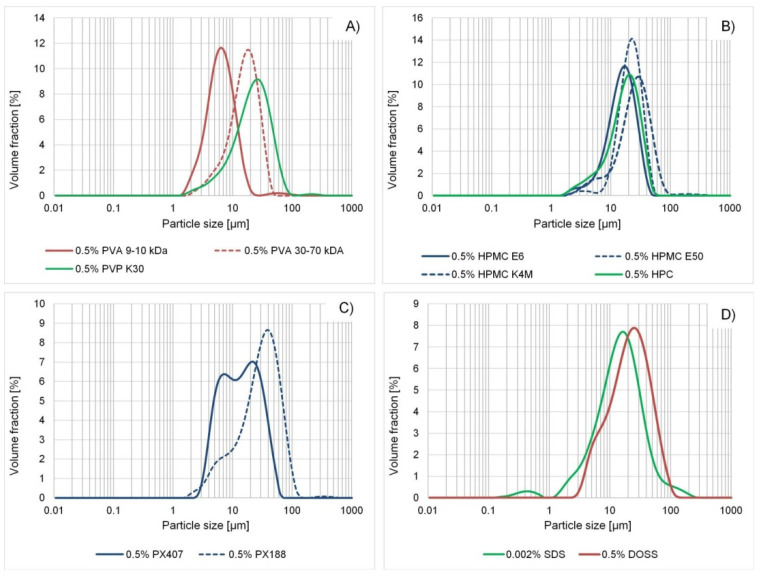
(**A**–**D**) Particle size distribution curves of cilostazol precipitated from 10% DMSO with different single stabilizers during stabilizer screening stage.

Among the tested single stabilizers, evidently the most promising results were produced with the application of PVA 9–10 kDa, i.e., monomodal distribution of microparticles at d90 = 12.10 µm (Figure 2 and Figure A3). For the same chemical type of a polymer, a correlation could be observed between its MW and precipitated particle size (see Appendix A). 

In particle size reduction and stabilization of nanosuspensions, combinations of different stabilizers are often found to be beneficial, for example due to supporting steric stabilization mechanisms with electrostatic repulsion [21]. We therefore checked whether the addition of nonionic or ionic surfactants to 0.5% PVA as the main polymeric stabilizer could further reduce CIL PSD; combinations of PX with anionic surfactants without any polymer stabilization were also evaluated. However, none of the tested combinations produced smaller PSD than PVA 9–10 kDa used as the only stabilizer. On the contrary, in general, the observed particle sizes seemed to be intermediate between those achieved with the respective single stabilizers (Figure 2). 

In the present study, PVA was therefore found to be the most promising stabilizer for further optimization based on comparatively the smallest size of precipitated microparticles. On the other hand, the descriptions of cilostazol nanosuspensions in the available literature do not report PVA use. Instead, among the used polymers, surfactants and their combinations, the following are mentioned: HPC+DOSS [14], low substituted MC+DOSS [15,16], Poloxamer [18,24], HPMC [28], HPMC+Tween 80 [17], and SDS (+Tween 80 or Kolliphor RH40) [19]. The current results apparently do not confirm the optimal potential of HPMC, HPC or PX for the particle size reduction of cilostazol. However, this discrepancy can be explained by the differences of processing methods employed, as the quoted works rely on top-down nanonization techniques (see Section 1) and it has been confirmed that a stabilizer’s efficiency may be different between mechanical comminution and bottom-up approaches [33,34]. 

In the current study, the advantage of PVA (9-10 kDa, 80% hydrolyzed) over other tested stabilizers is evident. A likely explanation for this affinity to cilostazol is favorable hydrogen bonding, since CIL molecule has 5 acceptor sites and 1 donor site, and most hydroxyl groups in PVA are donors [35,36]. On the other hand, other tested stabilizers either have only acceptor sites (PVP, PX, SDS, DOSS) or potential for both accepting and donating hydrogen atoms (HPMC, HPC) [37]. This might also explain why particle size reduction was less effective for LASP with stabilizer combinations–the interactions between PVA and acceptor stabilizers in AS solution were most likely strong enough to hinder efficient hydrogen bonding with cilostazol molecules. A very similar situation of PVA being the best stabilizer in LASP has been recorded for fenofibrate, another lipophilic molecule with multiple hydrogen acceptors [36]. For detailed discussion on relationships between particle size and stabilizer properties, see Appendix A.

### 2.3. LASP–Study of Factors and Optimization: Influence of Drug Concentration, Stabilizer Amount, Mixing and Feeding Speed

After selecting the solvent, S/AS ratio, and stabilizer as the formulation parameters with the best potential for generating small cilostazol particles/nanoparticles (Section 2.1 and Section 2.2), in depth precipitation process characterization and optimization was performed according to Design of Experiment (DoE) principles, where only quantitative variables were included (for design details, see Section 3.4). The results of face-centered central composite design study are presented in Table 1. The effects of four factors were statistically analyzed, i.e., cilostazol concentration in the solvent phase, solvent phase flow rate, the ratio of PVA to CIL and mixing speed.

Even though all the parameters describing CIL PSD were analyzed, the main response of interest was the value of d90 as characterizing the majority of the particle population. Although at first models were built including and excluding statistically significant effects with reasonable R^2^ values of 0.91–0.92, they could not be considered as sufficient because the assumption of homoscedasticity was not fulfilled (residuals tended to increase with higher observed d90 values). In order to correct this, the dependent variable was subjected to logarithmic transformation to stabilize the variance of residuals [38], based on the suggestion of Box-Cox transformation (λ = −0.05). In other words, for the purpose of further model refinement, the response analyzed was not raw d90 value, but ln d90 instead. 

**Figure 3:**
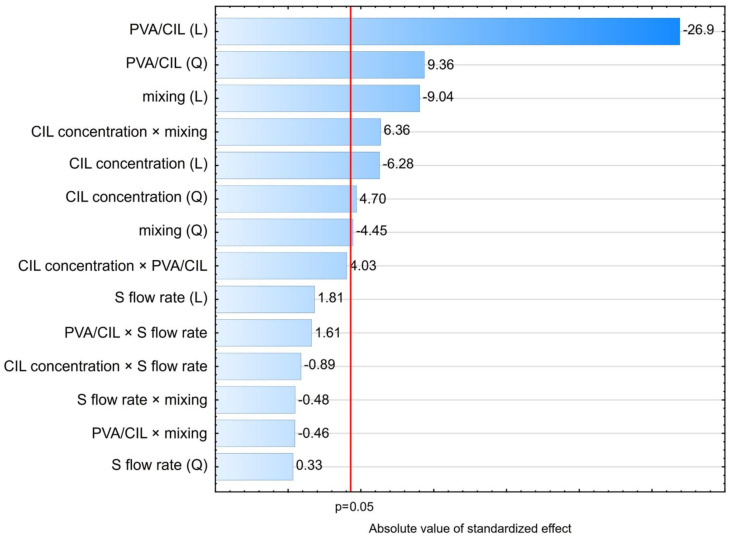
Pareto chart of the standardized linear (L), quadratic (Q) and linear interactions effect sizes of independent variables in liquid antisolvent precipitation (LASP) process studied according to central composite design plan. Bars above the red line denote statistically significant effects at *p* < 0.05.

According to ANOVA and Pareto chart (Figure 3), seven effects had a statistically significant (*p* < 0.05) influence on ln d90 value. According to this, *solvent phase flow rate (addition to antisolvent phase)* and its interactions with other variables did not affect particle size significantly. This result does not corroborate other works, where increase in flow rate was found to either decrease [39,40,41] or increase particle sizes [42,43]. There are many possible causes of this discrepancy. For instance, the cited reports relied on OFAT (one factor at a time) methodology instead of DoE and the variable was considered in isolation from other settings. It is also likely that the significance or size of this effect might be different for different ranges of particle sizes, being more pronounced for nanoparticles described in these reports than for the present microparticles. Finally, the contradictory character of the literature findings strongly suggests that the effect of solvent phase addition speed might be system-specific and play out differently for different combinations of drugs, solvents, stabilizers, and mixing equipment conditions. 

To both refine and simplify the model of the relationship between independent variables and ln d90, these statistically insignificant effects were eliminated. The final model equation was the following:

ln d90 = −0.02001 × CIL concentration + 0.0001 × (CIL concentration)^2^ − 3.61637 × PVA/CIL + 1.77473 × (PVA/CIL)^2^ + 0.00205 × mixing − 0.000003 × mixing^2^ + 0.00001 × CIL concentration × mixing + 4.18735 (3)

**Figure A4:**
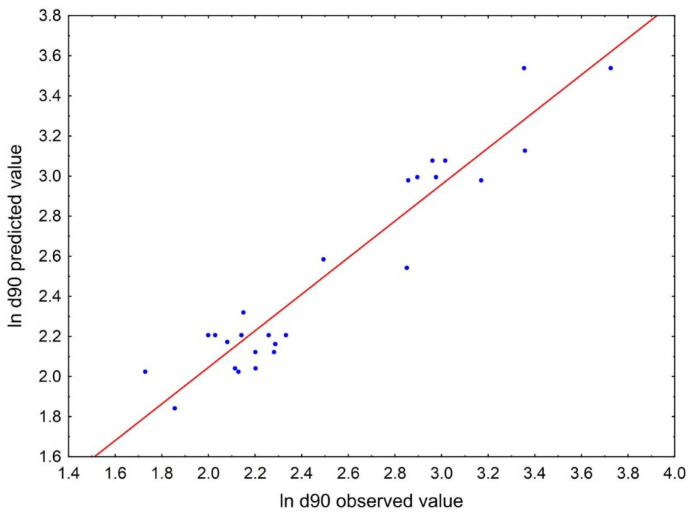
The correlation between observed and predicted ln d90 values by the final model (Equation (3)) for LASP parameters developed according to central composite design experiments.

The model was characterized by reasonably high coefficient of determination (R^2^ = 0.91, R^2^adj. = 0.88) and correlation between observed and predicted ln d90 values (Figure A4, Appendix B). It also satisfied other diagnostic criteria and thus was treated as the target equation for characterization and optimization of LASP process. 

**Figure 4:**
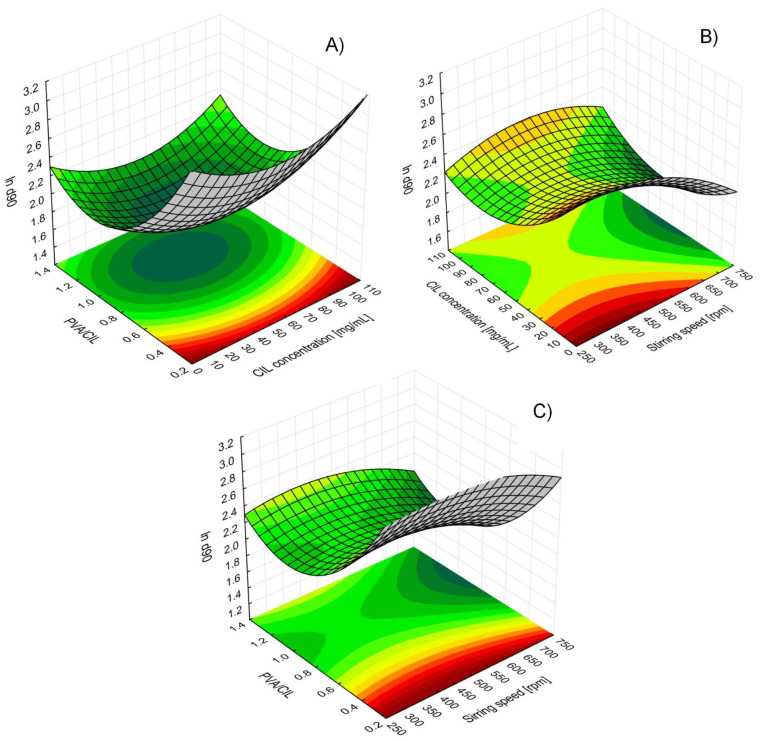
Response surface maps (RSM) for LASP with ln d90 as response variable. (**A**) PVA/CIL vs. CIL concentration at stirring = 750 rpm; (**B**) CIL concentration vs. stirring speed at PVA/CIL = 0.75; (**C**) PVA/CIL vs. stirring speed at CIL concentration = 55 mg/mL. Red colored areas correspond to the settings where highest values of ln d90 are achieved, while green color with increasing intensity - to the lowest values.

As can be seen from the model equation and response surface maps (RSM, Figure 4), the three remaining LASP process variables affect ln d90 values in a nonlinear way, and interaction between cilostazol concentration in the solvent phase and mixing speed is significant, which means that the setting of one variable modifies the influence of the other.

Undoubtedly, the deciding LASP parameter with the strongest effect on particle size is the *amount of PVA as stabilizer relative to the amount of cilostazol in the system* (Figure 3), whose linear negative effect is modified by positive quadratic effect. RSM analysis of the curvature (Figure 4A,C) reveals that d90 initially decreases with increasing PVA amount, reaching a minimum at PVA/CIL ratio of 1.0 most likely owing to optimal surface coverage with the stabilizer. A further increase in PVA amount leads to secondary particle size enlargement due to possible viscosity increase, diffusion hindrance or polymer bridging, which is not uncommon both in bottom-up [44,45,46,47] and top down processes [48]. A similar RSM curvature shape and effects direction can be observed for *CIL concentration in the solvent phase* (Figure 4A,B). Initially, with higher cilostazol feed concentration, the precipitated particle size decreases, as expected according to higher supersaturation and nucleation rate. However, a further increase in concentration results in larger particles, which may be attributed to agglomeration and particle growth competing with nucleation under high supersaturation conditions. At higher feed levels, cilostazol molecules are available to be readily incorporated onto growing crystal surfaces, which has also been described [40,44,49].

The main linear effect of *mixing speed* is the third strongest (Figure 3). As expected [39,43,45], its negative sign along with negative sign of quadratic effect mean that precipitated particle size is reduced with increased speed, which can be explained by enhanced macro- and micromixing and mass transfer between S and AS phase, translating to higher nucleation rates [20]. However, the influence of stirring speed is modified by its positive interaction effect with CIL concentration in solvent phase (Figure 4C). High feed concentrations should be therefore avoided when applying fast stirring, as this might possibly increase the occurrence of molecular and particulate collisions and cause the growth phenomena to prevail over nucleation, which also has been observed in LASP [46]. 

As evident from central composite design plan results (Table 1) and RSM (Figure 4), simple liquid antisolvent precipitation process was unable to produce cilostazol nanocrystals. In addition, desirability profiling or point predictions based on the model equation even with extrapolated settings revealed that no realistic combination of considered independent variables could result in particles with d90 < 1 µm. In a way, this confirms the few reports where supercritical antisolvent precipitation or LASP without modifications as a sole process resulted in the formation of CIL microparticles [24,28], even though successful production of nanosuspensions by bottom-up processes with simple setup has been described for several other drugs [36,41,42,43]. 

Therefore, LASP optimization in the current study was aimed at identifying the values of processing factors whose application would result in the smallest achievable d90 of microparticles for further development via precipitation-sonication (for validation of prediction experiments see Appendix B). Based on these results, therefore, in the next investigation stages PVA/CIL ratio = 1.0 and mixing speed = 600 rpm were fixed. 

### 2.4. Sonication–Study of Factors and Optimization

#### 2.4.1. Preliminary Sonication Studies: Moment of Ultrasound Activation, Sonication Pattern, Initial Temperature

Before systematical investigation of the quantitative influence of sonication parameters on cilostazol PSD, preliminary studies were carried out to choose the most promising setup by comparing the effect of qualitative settings: moment of ultrasound application (during vs. after precipitation), sonication pattern (continuous vs. intermittent) and initial antisolvent temperature (for details and rationale see Section 3.5.1). At this stage, the following parameters were kept constant: CIL concentration in the S phase (55 mg/mL), ultrasound (US) amplitude (60%) and sonication time of 25 min, to reflect the central values planned for the next stage of investigation (Section 2.4.2). 

The results of preliminary qualitative sonication study (Table 2) may be considered as somewhat surprising. Under constant ultrasound treatment at the same temperature conditions (setup 1 vs. 3 and 2 vs. 4) there was either no appreciable difference between *concomitant insonation during S phase addition and separated precipitation-sonication*, or the difference was in favor of two-step processing. It was expected that the application of ultrasound during the mixing of solvent and antisolvent would result in smaller particles according to the mechanisms improving nucleation rates, i.e., cavitation and bursting of air bubbles, which increases liquid velocities and improves micromixing. Sonication during phase mixing also is known to cause localized temperature drop resulting in rapid supersaturation, therefore effectively reducing metastable zone width and inducing nucleation [50,51]. Contrary to these expectations, sonication during precipitation did not provide any advantage, although it is difficult to attribute this to a particular cause. Possibly the 60% ultrasound amplitude of the particular processor and probe did not improve micromixing to a degree that would markedly induce nucleation sooner than under magnetic stirring and at the sufficient supersaturation achieved with 55 mg/mL feed concentration.

Interestingly, to the authors’ knowledge, no published study on the application of ultrasound for nanonization purposes has thus far compared the variants of concomitant vs. separated sonication. It has been found that for crystallization of large particles, the moment of ultrasound activation within a few minutes after solvent-antisolvent mixing influences crystal size, where reduction is effective only when sonication is introduced during initial stages of crystal growth, although the exact time seems to be system-specific and varies in the reports from 20 s [52], 60 s [53] to 120 s [54]. However, due to different research purposes, setup (including short sonication time in the order of seconds) and particle sizes of several tens or hundreds of microns in the cited works, these findings do not seem to relate directly to the present study, especially considering the fact that in two-step LASP+sonication variant, care was taken to apply ultrasound as soon as possible after phase mixing and precipitation was complete. 

Another unexpected result of the preliminary study was the *effect of initial AS temperature*. In numerous reports, reduction in processing temperature has resulted in smaller particles due to increased supersaturation or medium viscosity inhibiting particle growth [55,56,57,58,59] or affected the type of the crystallized (pseudo)polymorph [60]. This was not the case in the present study, where lower temperature reduced particle size only slightly and was related to worse repeatability for continuously insonated samples. In the case of intermittent sonication, decreased temperature caused about twofold increase in particle size, which suggests that at relatively higher supersaturation when compared to room temperature, pauses in ultrasound application enabled excessive growth, not compensated for by the total time of insonation. Additionally, there were no correlations at all between d90 value and any registered temperature values or temperature increments, the initial AS temperature, temperature after precipitation, or the final value after sonication. 

The comparison of *continuous and intermittent sonication* revealed no advantage of pulsed processing at room temperature, and even a disadvantage at lower temperature. To the authors’ knowledge, studies on nanosuspensions described in the publicly available literature focus mostly on one chosen mode and in one found example there was no significant difference between the variants [61]. However, the present results are in agreement with the case studies of large particles crystallization reviewed by Ruecroft et al., according to which continuous insonation reduces particle sizes, while short ultrasound bursts favor crystal growth [23]. 

To summarize, based on the preliminary studies, the following setup was chosen for further investigation according to DoE: two-step LASP+sonication in continuous mode with initial AS temperature 25 °C (No. 4 in Table 2). The fact that precipitation followed by insonation as a separate step proved to be useful in particle size reduction tentatively indicates that the main mechanism involved is not reduced metastable zone width and faster nucleation. Instead, it appears to be related to sonofragmentation due to cavitation-induced shockwaves affecting the existing particles surfaces, breaking them and initiating secondary nucleation sites [62,63]. Moreover, the sonication step after LASP supports deagglomeration and may serve as an annealing factor, enhancing the adsorption and reordering stabilizer coverage on crystal surfaces [55,64]. 

#### 2.4.2. LASP+Sonication Study of Factors Using DoE: Influence of Drug Concentration, Sonication Time, and Amplitude

After identifying the qualitative setup which results in the smallest achievable cilostazol PSD in preliminary studies (Section 2.4.1), in depth precipitation-sonication relationships were characterized with DoE to study the effect of feed concentration and ultrasound time and amplitude. Although the effect of CIL concentration in solvent phase had already been investigated during LASP optimization, this variable was again included in the experimental plan, because modified nucleation conditions and increased molecular mobility under ultrasound action might change the effect magnitude, and interactions with sonication amplitude or time need to be considered. 

**Figure 5:**
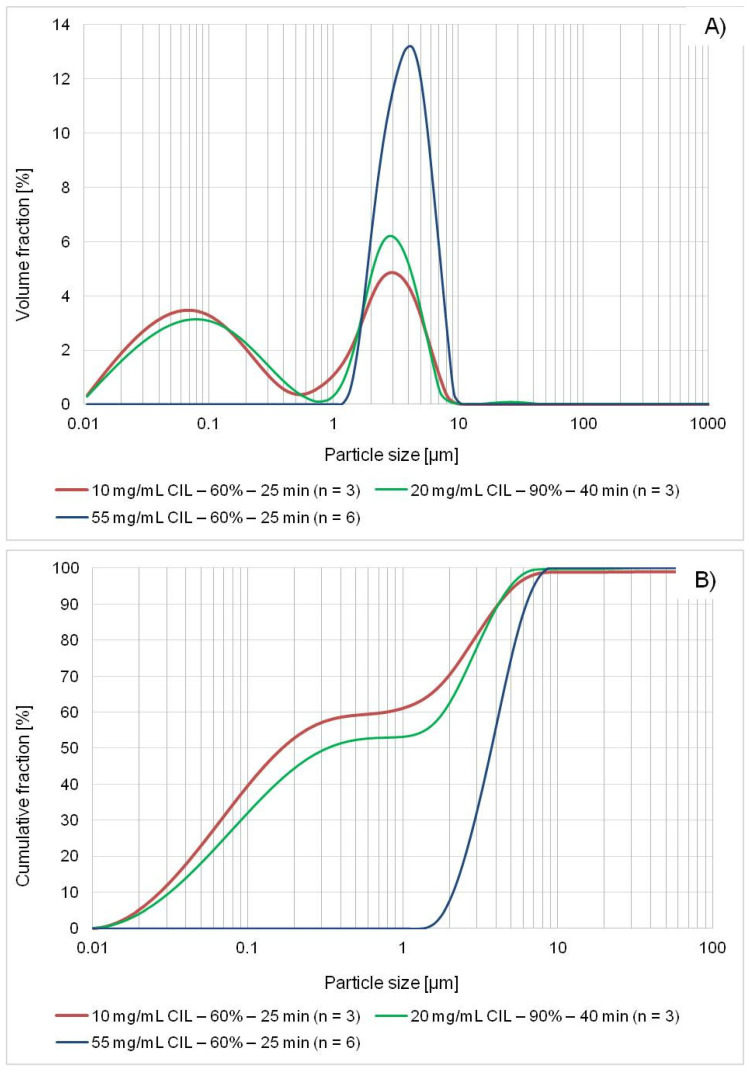
A comparison of monomodal microparticle population and bimodal nano- and microparticles obtained at different independent variable settings of LASP+sonication studied according to central composite design ((**A**) frequency size distribution, (**B**) cumulative distribution).

The results of central composite design experiments for precipitation-sonication are given in Table 3. The majority of tested independent variable settings showed little improvement over simple LASP, as they resulted in a single population of microparticles. However, two of the tested combinations (feed concentration 20 mg/mL-amplitude 90%-40 min, and 10 mg/mL-60%-25 min) produced bimodal distributions of micro- and nanoparticulate fraction (Table 3, Figure 5). This is reflected in their d10~30 nm, d50~200–400 nm and d90~4 μm values, although it must be noted that description of polymodal particle populations with single PSD parameters must be cautious and it is difficult to make direct quantitative comparisons of such results with straightforward PSD parameters of monomodal samples. With this caveat, it was nevertheless deemed useful to statistically analyze DoE results and d50 value was the primary response of interest in this case, as the dependent variable which reflects differences between nano- and microsuspensions. 

**Figure 6:**
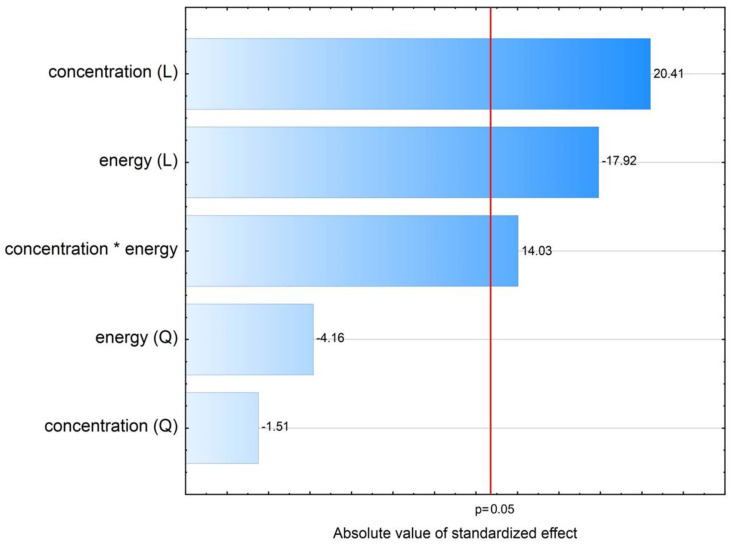
Pareto chart of the standardized linear (L) and quadratic (Q) effect sizes and interactions in precipitation + sonication process studied according to central composite design plan. Bars above the red line denote statistically significant effects at *p* < 0.05.

The process of model development for d50 values is detailed in Appendix B. Briefly, the results of axial plan points where CIL feed concentration was set at 10 mg/mL needed to be excluded from model analysis. Moreover, sonication time and amplitude were substituted with a new single compounded variable: ultrasound energy delivered to the system (Table 3). The effects sizes of the analysis according to this principle are presented in the Pareto chart (Figure 6). 

For further simplification and refinement, the statistically insignificant quadratic effects of cilostazol concentration and insonation energy were excluded from the final equation:

d50 = 0.001198 × CIL concentration − 0.000085 × energy + 0.000001 × CIL concentration × energy + 4.278373(4)

As a result, this was chosen as the definite model to describe the relationship between precipitation-sonication process variables and d50 value. Its diagnostic criteria were acceptable, with reasonable regression coefficient (R^2^ = 0.89, R^2^adj. = 0.88), insignificant lack-of-fit and normal distribution of residuals according to Shapiro-Wilk test. 

**Figure 7:**
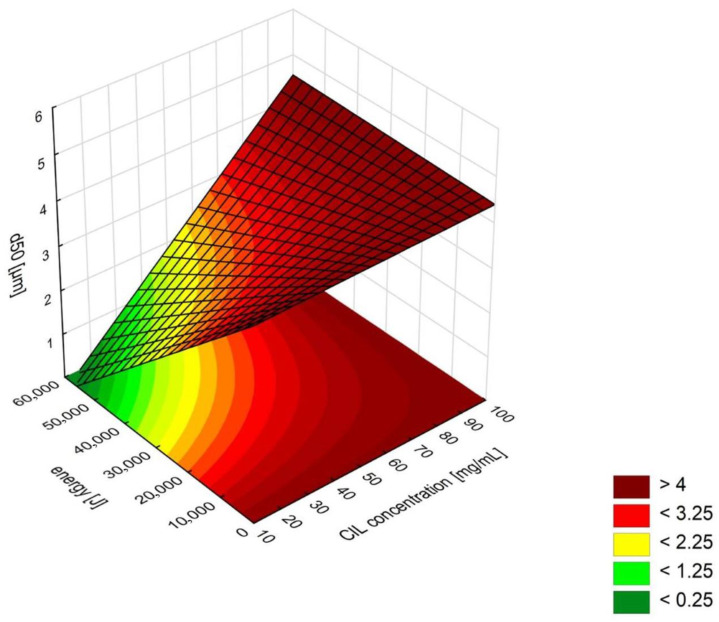
Response surface map for precipitation-sonication process with d50 value as response.

The general interpretation of sonication effects on d50 value is straightforward in this case. The positive sign of CIL concentration, which is the strongest effect, denotes that with increasing feed load particles tend to be larger. The positive interaction sign with ultrasonic energy suggests that at high concentrations and simultaneous higher amplitudes or longer insonation times, particle growth prevails, possibly due to increased collisions frequency and condensation. This is in agreement with many sonoprecipitation studies [55,58,65], although depending on the API and its concentration range, an initial particle size decrease with secondary increase has also been reported [57,59,66]. The most favorable settings for size reduction are therefore low cilostazol concentration in the S phase and high amplitude and duration of ultrasound application, possibly to provide sufficient energy input for thorough particle sonofragmentation and effective stabilizer adsorption (Figure 7). This is expected and confirms numerous findings regarding the influence of ultrasound power/amplitude and time [55,56,57,58,59,64,67,68]. 

**Figure A6:**
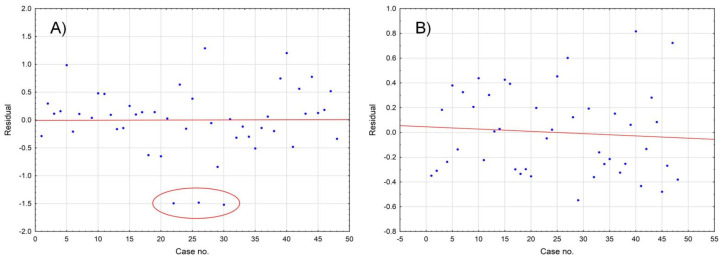
Distribution of residuals according to case No. corresponding to the order of runs in precipitation-sonication central composite design (Table 3): (**A**) According to initial full model built including all the plan points. The outlier residuals resulting from the inclusion of axial points at CIL concentration = 10 mg/mL are marked in red. (**B**) According to the final model built with the exclusion of axial points producing outlier residuals.

It must be noted that the developed final model, while statistically sound, can serve only as a tool for the characterization of LASP-sonication process and general evaluation of factor effects, but not for direct optimization. Its predictive power is evidently limited, since model building required the exclusion of results generated at some axial points. In other words, the detected mathematical relationships are valid only for feed concentration ranges down to 20 mg/mL, as the results with 10 mg/mL input could not be predicted reliably (Figure A6, Appendix B). Any calculations of the d50 value for 10 mg/mL-60%-25 min (~18,000 J) (settings in runs 41, 9, 25 in Table 3) would severely overestimate the observed result at ~2 µm instead of 200–300 nm, including the non-final equations explored throughout model development. 

This might be partly related to the limited accuracy of d50 value as a measured response for bimodal populations as discussed earlier. However, given the fact that bimodal particle distributions obtained for runs 26, 36 and 4 (at 20 mg/mL) were successfully included in the model building, this reason would be considered secondary. Apparently, there exists a certain value of CIL concentration in the solvent phase below which the quantitative relationship between LASP-sonication process parameters and d50 value is different than for higher concentration ranges. This is also evident from the fact that similar PSD with nanoparticulate fraction was achieved at a relatively small difference of cilostazol concentration (10 vs. 20 mg/mL), but for the lower feed setting, markedly lower energy input was required for partial nanonization (18,000 J corresponding to 25 min sonication at 60% amplitude vs. 55,000 J at 90% and 40 min). Therefore, it must be concluded that a single equation derived from linear regression principles is unable to model and predict the relationship between precipitation-sonication parameters and nano/microsuspension particle size. To the authors’ knowledge, in the publicly available literature on sonoprecipitation employing DoE such situation has not yet been reported, possibly because other types of factors than sonication energy and feed concentration are mostly evaluated with this methodology [47,55,57,59,61,67,69,70,71,72,73,74]. One exception is the work of Sharma et al., who analyzed the effect of drug concentration, injection rate and sonication time using Box-Behnken design with results differing from the present report, i.e., interaction effect was insignificant and both concentration and time displayed negative linear effects modified by positive quadratic effects [75]. 

The presented situation contrasts with the results of DoE for simple LASP without ultrasound, where a reasonably predictive model was found and validated (Section 2.3), enabling process optimization. The cause of this difference is most likely related to the different effect of CIL concentration in antisolvent precipitation alone and when combined with sonication, as there is clear discrepancy between value ranges enabling the smallest possible particle size for the particular process (Figure 4 and Figure 7). This might possibly be attributed to cavitation phenomena inducing higher supersaturation than would be generated at a the same cilostazol concentration without ultrasound, which shifts the optimal nucleation and reduced particle growth to lower setting ranges. 

#### 2.4.3. LASP+Sonication Optimization

To summarize, DoE results analysis due to lack of predictive potential could not directly identify parameter settings which would produce nanosuspension. Therefore, optimization of LASP+sonication was carried out based on empirical approach, guided by the characterization results of central composite design plan. Since low CIL concentration levels were the most promising, yielding at least partially nanosized material, and since ultrasound energy increase reduced the particle size, optimization consisted of point studies at 10 and 20 mg/mL concentrations and 90 and 100% amplitude, with sonication time increased experimentally. The aim of this optimization study was to find parameter settings which would eliminate the separate population of microparticles, reducing d90 value to ~1 µm or lower. As DoE revealed different nucleation behavior at lower ranges of CIL concentrations, the influence of precipitation variant (single step vs. two-step) and initial AS temperature was reevaluated at these new settings for comparison purposes with preliminary study at 55 mg/mL (Section 2.4.1).

The results of six optimization study settings (n = 3) with amplitude fixed at 90% and time at 40 min (resulting in energy input of 55,000 J) are collected in Table 4. The advantage of separate LASP and sonication processes over concomitant sonoprecipitation was confirmed. Comparison of the results of setup No. 6 with the results of DoE runs 26, 36, and 4 (Table 3), where the only difference was the initial AS temperature, indicates that at 20 mg/mL lowering the temperature value is detrimental and apparently supersaturation is excessive. However, the system showed the opposite at 10 mg/mL (setup 3 vs. 4), where the lower temperature value was more beneficial for particle size reduction in nanoscale. This observation reinforces the complex interplay of factors affecting supersaturation, nucleation, and growth, which makes it difficult to model exactly their effects on particle sizes. 

**Figure 8:**
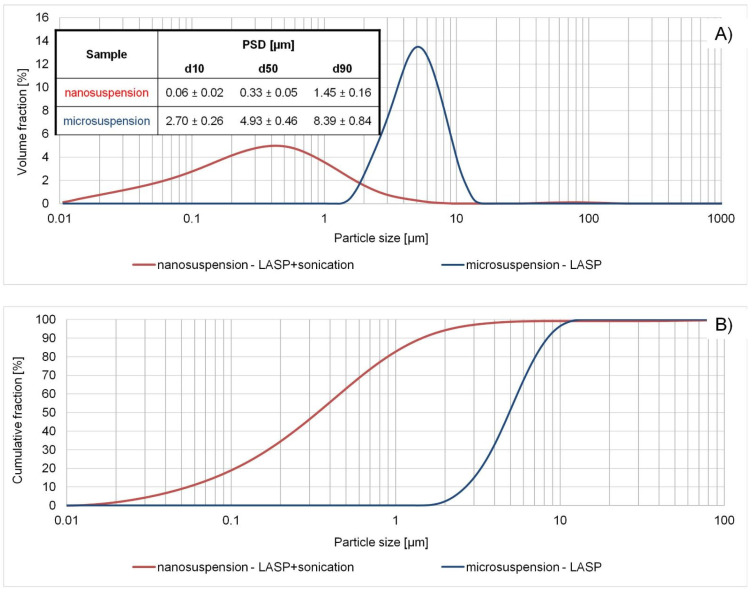
Comparison of particle size distributions ((**A**) frequency distribution, (**B**) cumulative distribution) of optimized cilostazol nano- and microsuspensions (mean ± SD, n = 3).

Finally, combination No. 3 was chosen for last stage optimization, where the samples were sonicated at 100% amplitude for 45–70 min. Owing to this systematic approach, nanosuspension of monomodal particle size distribution was successfully produced and further characterized to compare with microsuspension (Figure 8). Although its d90 = 1.54 µm is technically above 1 µm, the optimized system still qualifies as a nanosuspension, since according to some definitions a product can be classified as drug nanocrystals with d50 < 1 µm, d90 < 2.5 µm or d99 < 5 µm [76,77,78]. With d50 = 0.33 µm and over 80% of particle population in nanoscale (Figure 8B), the developed material clearly fulfills these criteria.

To summarize, the optimal LASP-sonication factor settings for the generation of nanosized particles were the following: DMSO as solvent phase, PVA aqueous solution as antisolvent, S/AS 10/90, cilostazol concentration in S: 10 mg/mL, PVA/CIL ratio: 1.0, stirring during precipitation: 600 rpm, sonication amplitude: 100%, sonication time: 60 min, corresponding to the energy of ~93,000 J. In this way, successful production of cilostazol nanocrystals using a bottom-up method was demonstrated and the advantage of insonation for achieving nanosized particles was proved where simple liquid antisolvent precipitation alone was insufficient, generating only microparticles. 

In the publicly available literature, one example of cliostazol nanonization with bottom-up technique has been described by Choi [29]. Coincidentally, in the cited work, the same solvent and S/AS ratio were selected as optimal, i.e., 10% DMSO. Precipitated particles were immediately subjected to high-speed homogenization or probe sonication, where only ultrasound of 200 W and 3 min was able to produce nanoparticles, with minimum size 553 ± 183 nm at 4 mg/mL drug concentration and 4 °C. However, due to several methodological differences, it is difficult to directly compare the results and explain discrepancies of the current study with Choi’s report. In our case, the influence of temperature was not evident, while in the abovementioned publication, particle size increase with temperature was observed. This may be due to the atypically broad range studied in the cited work (4–80 °C), where it is likely that at higher levels the supersaturation degree driving particle nucleation might be lost. A close inspection of the results reported by Choi also reveals that between 4 °C and 25 °C the size difference is minimal and within standard deviation (713 ± 232 vs. 841 ± 443 nm) [29]. This would corroborate our findings that for liquid antisolvent precipitation of cilostazol, temperature within certain range is not a factor of critical importance. 

Considering the same solvent and S/AS chosen, a major difference between the present work and Choi’s report is the drug concentration range that we studied: 10–100 vs. 1–4 mg/mL. This results in markedly different supersaturation conditions, which translated in the cited work to the highest level of concentration yielding the smallest particles [29]. For the lack of common ground in this case, comparative speculation seems unwarranted, but the current study fills the knowledge gap by addressing thoroughly the relationship between particle size and cilostazol feed concentration practically in the whole feasible range, up to the point slightly below saturation solubility of CIL in DMSO. Moreover, it successfully demonstrates that cilostazol nanonization is possible at 10 mg/mL, which could be beneficial for higher drug loads of the nanosupesnion. The difference in the concentration ranges most likely is also the source of disparity in optimal sonication time (60 vs. 3 min), along with equipment properties and ultrasound power (130 vs. 200 W). 

The major difference between the present work and the process described by Choi is the presence or absence or stabilizers. Here, once PVA as the optimal stabilizer was selected during the screening studies, it remained for LASP optimization, and once its optimal amount was identified, it was fixed for further process development. Since the qualitative advantage of PVA in particle size reduction was evident even with other parameters far from optimization, no attempts were made to explore processing without stabilizer, considering the fact that successful generation and especially stabilization of nanosuspension without polymer and/or surfactant addition is rare [79]. This relates to another major methodological difference between the two studies. Here, PSD was measured for freshly precipitated nanosuspension, whose stability in liquid form was evaluated (Section 2.5.1), while Choi reports immediate collection of the nanocrystals via centrifugation or filtration, so no data on original suspension size or its stability is given and there is no evaluation of cilostazol’s micro/nanosuspension size evolution in time [29]. Moreover, the employed particle size measurement techniques (laser diffraction vs. dynamic light scattering) are based on different principles, and the parameters describing particle size distribution (volume based d10–d90 vs. hydrodynamic size z-ave) cannot be directly compared, especially given the fact that DLS technique is not geared towards detecting larger populations which could accompany nanoparticles. Finally, Choi’s work does not report on dissolution behavior of sonoprecipitated cilostazol nanocrystals [29]. Therefore, the current study aims to address this gap and fully characterize bottom-up processed CIL nanosuspension. 

### 2.5. Characterization of Micro- and Nanosuspensions

#### 2.5.1. Physical Stability and Process Efficiency

Precipitation process efficiency calculated from the concentration of dissolved cilostazol remaining in mother liquor can be considered as satisfying at 98–99%, both for simple LASP and combined with sonication. Interestingly, CIL concentration in the filtrates reached 17 ± 2 µg/mL, almost twice as high as the drug’s equilibrium solubility in 10% DMSO (8.8 ± 0.06 µg/mL), which can be explained by the ability of PVA to inhibit precipitation and stabilize supersaturation to a certain degree [80]. 

**Figure 9:**
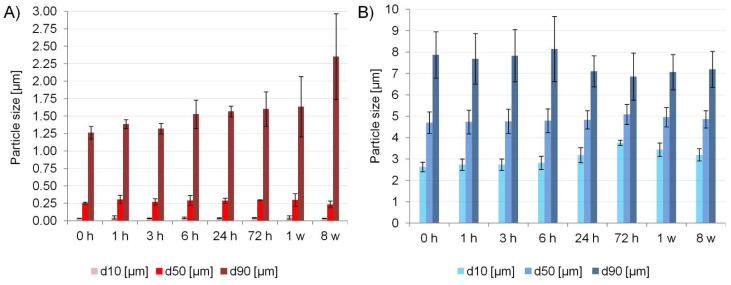
Particle size distribution stability of nanosuspensions (**A**) and microsuspensions (**B**).

Both micro- and nanosuspensions proved to be stable over a 1-week period, without obvious signs of Ostwald ripening, and in the case of microcrystals the PSD even tended to be narrower over time. After 2 months, microsuspensions retained their size, while some growth occurred for nanosuspesions as reflected in their d90 value. Nevertheless, the majority of particles remained nanosized (Figure 9). Particle size stability is a critical quality attribute of nanosuspensions as a prerequisite for retaining improved dissolution rates over gastric and intestinal transit times when administered orally. Moreover, the steady PSD confirms the suitability of PVA as the optimal stabilizer for bottom-up processed cilostazol. 

#### 2.5.2. Solid State and Morphology

**Figure 10:**
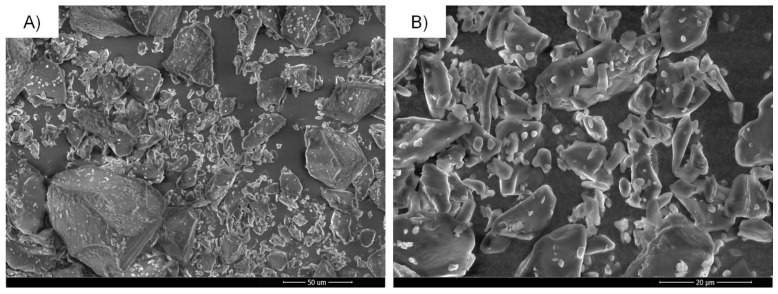

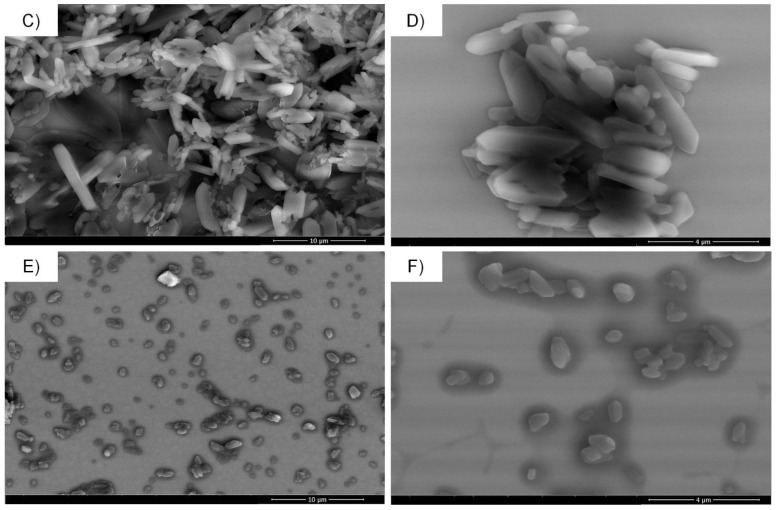
Scanning electron microscopy (SEM) images of raw, unprocessed cilostazol powder (**A**,**B**), microcrystals (**C**,**D**) and nanocrystals (**E**,**F**). (**A**) magnification 1500×, scale bar 50 µm; (**B**) magnification 5000×, scale bar 20 µm); (**C**,**E**) magnification 10,000×, scale bar 10 µm; (**D**,**F**) magnification 30,000×, scale bar 4 µm.

**Figure 11:**
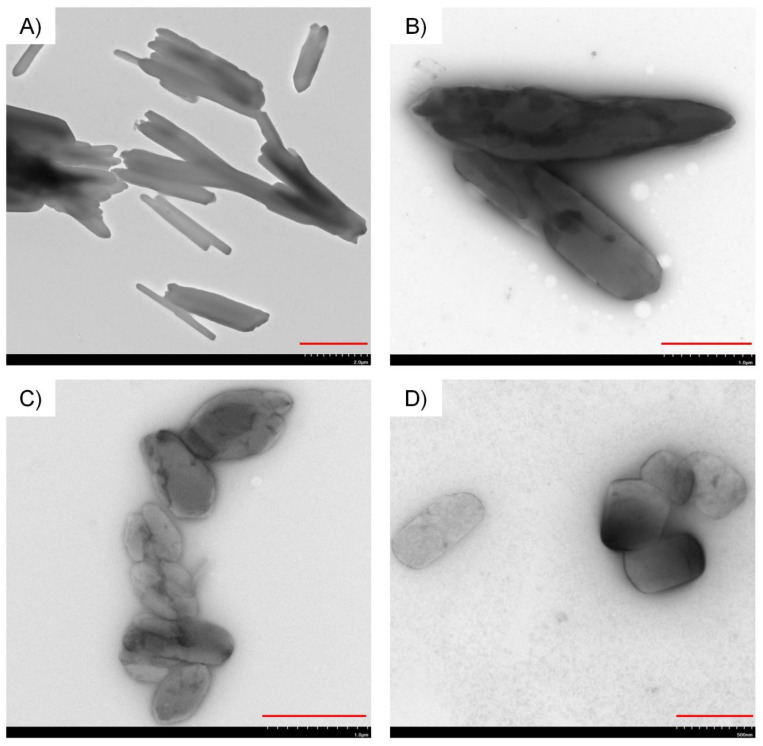
Transmission electron microscopy (TEM) images of cilostazol microcrystals (**A**,**B**) and nanocrystals (**C**,**D**). (**A**) magnification 22,000×, scale bar 2 µm; (**B**) magnification 62,000×, scale bar 1 µm; (**C**) magnification 70,000×, scale bar 1 µm; (**D**) magnification 110,000×, scale bar 500 nm.

**Figure 12:**
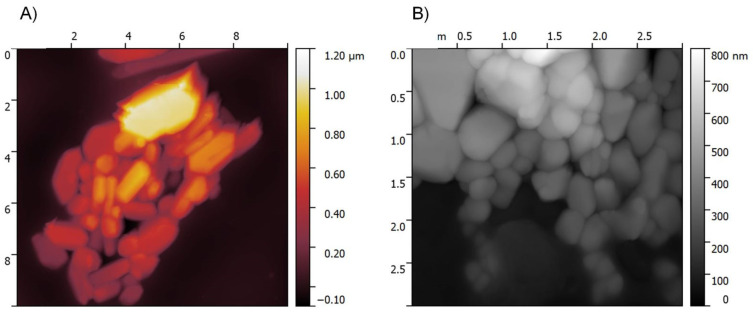
Atomic force microscopy (AFM) images of cilostazol microcrystals (**A**) and nanocrystals (**B**).

The particle morphology of raw, untreated cilostazol as the starting material, microcrystals and nanocrystals was compared with the use of three complimentary imaging techniques: scanning electron microscopy (SEM, Figure 10), transmission electron microscopy (TEM, Figure 11) and atomic force microscopy (AFM, Figure 12). This enabled a quick visual confirmation of differences in particle sizes between the three types of material, as well as an assessment of particle shapes and surface appearance. According to this, raw, unprocessed cilostazol powder consists of populations of smaller, granular particles and large angular and plate-like structures (Figure 10A,B). Precipitation in the presence of equal weight amount of PVA changed not only the particle size, but also the dominant crystal habit to more elongated, rod-like structures (Figure 10, Figure 11 and Figure 12), which has also been observed for recrystallized cilostazol [27,28]. Further processing with sonication induced changes not only in the reduced particle size, but also in morphology, as the nanocrystals were predominantly more regular, spherical, or granular in shape when compared to microcrystals (Figure 10, Figure 11 and Figure 12). The effect of ultrasound on crystal habit may likely be explained by preferential sonofragmentation and breakage at selected points of elongated microcrystals, together with efficient adsorption of PVA to newly exposed surfaces of broken microcrystals, preventing further directional growth [55,62,63]. It is interesting to note that compared to other reports on cilostazol processed with sonoprecipitation, the present nanocrystals appear more spherical. The difference might stem from the absence of stabilizers and shorter application of ultrasound in the works of Tari et al. and Choi [27,29]. 

**Figure 13:**
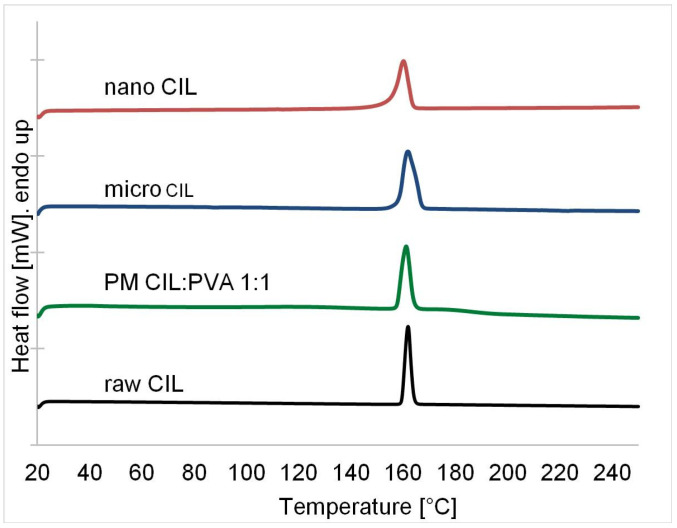
Differential scanning calorimetry (DSC) curves of unprocessed cilostazol, its physical mixture (PM) with PVA, and micro- and nanocrystals.

The changes of cilostazol crystal habit after precipitation and sonication were not related to any polymorphic transition, as revealed by differential scanning calorimetry (DSC) and X-ray powder diffractometry (XRPD). The only thermal event of importance on DSC curves is the endothermic peak corresponding to melting of the most stable crystalline form A at 161 °C for raw cilostazol (Figure 13) [27,32]. For the precipitated material, slight peak broadening can be observed due to the presence of PVA or minor impurities. Nevertheless, the endotherm’s location remained at 160–161 °C. No desolvatation exotherms can be seen, which was confirmed by TGA measurements (data not shown), indicating no formation of cilostazol pseudopolymorphs when precipitated from DMSO. For micro- and nanocrystals, approximate degree of crystallinity was calculated as the ratio of their melting enthalpies (ΔH = 113.97 and 112.46 J/g, respectively) to the melting enthalpy of raw CIL (ΔH = 125.50 J/g). The values of 91% (microcrystals) and 89% (nanocrystals) confirmed that potential fraction of amorphous material next to polymorph A is negligible; moreover, the degree of crystallinity is likely to be underestimated due to the presence of adsorbed PVA in the collected samples.

**Figure 14:**
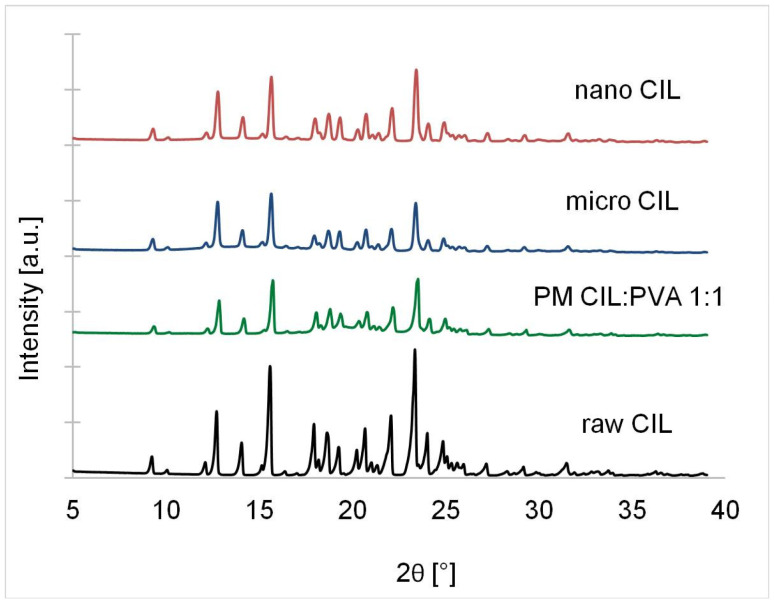
X-ray powder diffraction (XRPD) patterns of unprocessed cilostazol, its physical mixture (PM) with PVA, and micro- and nanocrystals.

The absence of polymorphic transition was confirmed with XRPD, where all the samples retained original peak positions (including characteristic peaks at 2θ values of 9.2, 14.0, 15.5 and 22.0) with intensity changes (Figure 14). The retention of the original and most stable crystalline form A agrees with other reports on cilostazol nanocrystals or reprecipitation processes [17,18,24,25,26,27,29], since appearance of form B and C in the work of Miao et al. is related to spray drying [28], while amorphization reported by Aghrbi et al. can be attributed to extrusion with excipients [19]. To the authors’ knowledge, so far, no publicly described crystallization process has been able to generate any polymorphic form other than A for cilostazol, unlike for many other compounds, where conditions such as antisolvent addition rate, mixing, temperature or presence of additives govern the resulting crystalline form [81]. 

**Figure 15:**
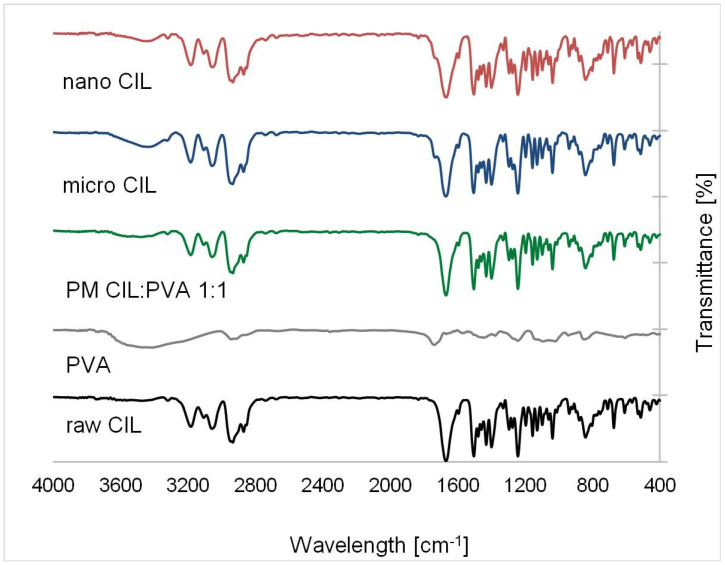
Fourier-transformed infrared (FTIR) spectra of unprocessed cilostazol, PVA, physical mixture (PM) and micro- and nanocrystals.

The chemical identity of cilostazol in every sample was also confirmed with Fourier-transformed infrared (FTIR) spectra (Figure 15), where characteristic bands are present (amide stretching at 3474, C=O stretching at 1669, N-H tetrazole bending at 1504, N=N tetrazole stretching at 1295 or C–O stretching at 1243 cm^−1^) [25,82]. No major evident band shifts were observed, although certain bands exhibited slight changes in intensities.

#### 2.5.3. Solubility and Dissolution Rate

**Figure 16:**
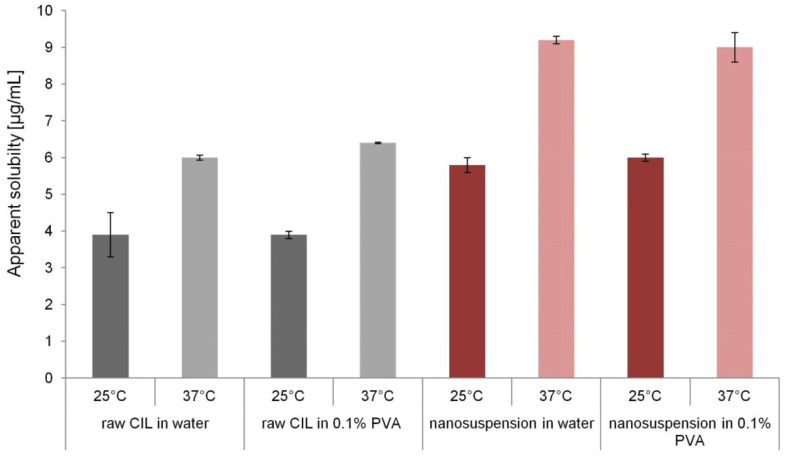
Apparent solubility (mean ± SD, n = 3) of unprocessed cilostazol and nanosuspension in water and in 0.1% PVA solution at 25 °C and 37 °C.

The results of apparent solubility studies are presented in Figure 16. Evidently, the nanosuspension’s solubility is significantly higher (*p* < 0.05) than cilostazol equilibrium solubility achieved by the raw material, regardless of the temperature (~6 vs. 4 µg/mL at 25 °C, and 9 vs. 6 µg/mL at 37 °C, i.e., increase of about 50%). For the respective samples, the presence of 0.1% PVA in the medium at its concentration corresponding to the composition of AS phase did not affect the CIL solubility when compared to water. It was also verified that DMSO present at max. 1.5% *v*/*v* in nanosuspension samples in solubility studies did not increase cilostazol equilibrium solubility (data not shown). Therefore, in the absence of polymorphic transitions, any apparent solubility advantage of nanosuspensions over unprocessed CIL can be attributed to the reduction of particle size to nanoscale. 

The measured equilibrium solubility value of 6 µg/mL at 37 °C is in agreement with the findings of Jinno et al. [14] and higher than those reported by Choi [29], Miao et al. [28] or Komasaka et al. [17] (3.5–4.5 µg/mL), which are likely underestimated due to insufficient shaking time for the coarse cilostazol to achieve equilibrium state. Regarding experimental measurements of apparent solubility for cilostazol nanoparticles, disparate results are presented in the literature. The concentration of ~9 µg/mL determined in the current study at 37 °C is reasonably close to that determined for 667 nm particles (9.8 µg/mL) by Miao et al. and much lower than that of 326 nm particles (23.1 µg/mL) [28]. However, in the cited work, spray-dried material was assessed, where the presence of cilostazol crystal forms B and C was also detected. Moreover, the reported literature value might be overestimated due to the use of 0.2 µm filters for the separation of nanoparticles, which are known to be inadequate in retention of smaller nanocrystal fractions despite suitable nominal cutoff [83,84]. Inefficient separation of nanoparticles from the supernatant during centrifugation may be the cause of atypically high solubility of 37.5 µg/mL reported by Choi (almost 10 times higher than the raw cilostazol’s value) [29]. 

In the current study, the experimentally determined increase of about 50% in cilostazol solubility due to nanonization is higher than the theoretical values calculated for CIL nanocrystals according to Ostwald-Freundlich equation (Equation (2)): 30–40% for d10~100 nm, 7–9% for d50~220 nm, 3% for d90~1 µm, as well as higher than the 6% increase for cilostazol nanoparticles calculated by Jinno et al. [14]. This situation is different from several reports on other drug nanosuspensions, where theoretical and experimental solubility increases (5–30%, depending on particle sizes) were in agreement [83,85,86]. The discrepancy between Ostwald-Freundlich predictions and experimental results in the current study probably might be related mostly to the fact that the captured elevated apparent solubility of nanosuspensions may be of kinetic, and not thermodynamic nature, i.e., a long-term supersaturation effect of dissolution process instead of increased saturation solubility of nanoparticles. Moreover, the current calculations of Ostwald-Freundlich equation are just a simplified approximation where single PSD values were incorporated into calculation. Since advanced modeling of the whole nanoparticles population behavior and their changing dimensions in time with progressing dissolution was outside the scope of this study, the presented comparison of theoretical and experimental solubility values must be treated with caution as a rough estimate. Nevertheless, the observed solubility increase seems to be more conservative and closer to the model when compared to the values reported for cilostazol nanocrystals by other authors [28,29]. 

**Figure 17:**
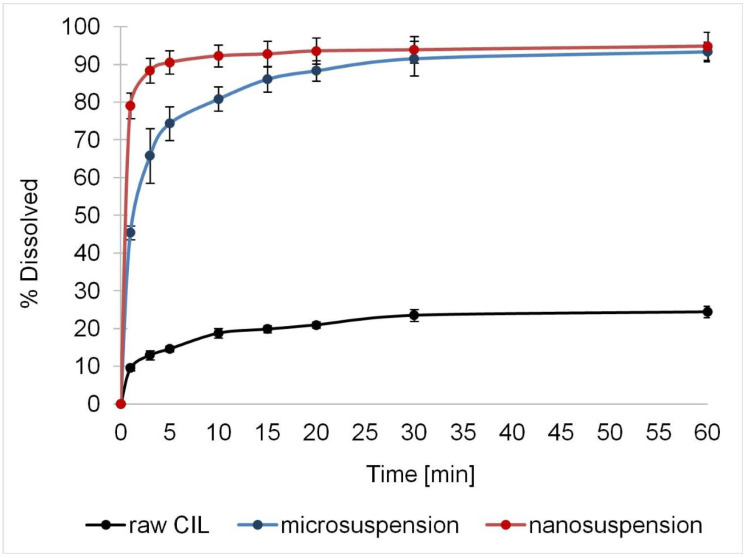
Dissolution profiles (mean ± SD) of unprocessed cilostazol (n = 6), microsuspension and nanosuspension (n = 7).

The dissolution profiles of raw cilostazol, microsuspension and nanosuspension are presented in Figure 17. The differences and rank order expected according to particle sizes were confirmed, with incomplete dissolution of coarse raw CIL (d90 = 75 µm) markedly slower than in the case of precipitated particles. Both micro- and nanosuspension dissolved completely in non-sink conditions and achieved a similar plateau level after about 30 min. Still, nanoparticles exhibited clear advantage in dissolution rate, as the process was finished within 3–5 min, as often observed for nanosized drugs, including cilostazol [14,17,85]. The dissolution of microparticles progressed more slowly and the two profiles must be considered as significantly different according to similarity factor value below 50 (f2 = 40.67). First order models were suitably fitted to describe the dissolution profiles (R^2^adj.= 0.90 for raw CIL, 0.96 for microparticles and 0.99 for nanoparticles, respectively), which confirms the validity of assumption of Noyes-Whitney dissolution kinetics. Significant differences (*p* < 0.05 according to ANOVA post-hoc test) were found for dissolution rate constants in descending order from nanocrystals to raw cilostazol, i.e., 1.958 > 0.543 > 0.292 min^−1^, respectively. These results therefore confirm the literature findings and the rationale for nanosizing of cilostazol for the improvement of its dissolution rate.

## 3. Materials and Methods

### 3.1. Materials

Raw cilostazol produced by Glenmark (Mumbai, India) was kindly gifted by Przedsiębiorstwo Farmaceutyczne Lek-Am Sp. z o.o. Vivapharm HPMC E6 (Hypromellose 2910) was donated by JRS Pharma (Rettenmaier Polska Sp. z o.o., Warsaw, Poland). Other materials were purchased commercially. P.a. grade glacial acetic acid, acetonitrile, N,N-dimethylformamide (DMF), dimethyl sulfoxide (DMSO) and methanol were obtained from Chempur (Piekary Śląskie, Poland) and poly(ethylene glycol) average MW 400 g/mol (PEG400) was obtained from Acros Organics (Thermo Fisher Scientific, Waltham, MA, USA). Poly(vinyl alcohol) MW 9-10 kDa 80% hydrolyzed (PVA 9–10 kDa), poly(vinyl alcohol) average MW 30–70 kDa 87–90% hydrolyzed (PVA 30–70 kDa), Kolliphor P188 (poloxamer 188, PX188) and Kolliphor P407 (poloxamer 407, PX407) were purchased from Sigma Aldrich (Merck Life Science Sp.z.o.o., Poznan, Poland). Hydroxypropyl methylcellulose HPMC E50 and dioctyl sulfosuccinate sodium salt 96% (DOSS) were obtained from Alfa Aesar (Ward Hill, MA, USA). Hydroxypropyl methylcellulose 86 kDa HPMC K4M, polyvinylpyrrolidone average MW 50 kDa (PVP K30), hydroxypropyl cellulose average MW 100 kDa (HPC) were obtained from Acros Organics and sodium dodecyl sulfate Ph. Eur. grade (SDS) was purchased from Pol-Aura (Dywity, Poland). 

### 3.2. Solvent Screening

Cilostazol equilibrium solubility in each pure solvent was determined by shake-flask method (IKA KS130 Control shaker, IKA^®^ Poland Sp. z o.o., Warsaw, Poland). Next, nearly saturated solutions in the respective solvents were mixed with water at ratios 5–80% (*v*/*v*). After shaking for 72 h at 25 °C cilostazol concentrations were quantified with a UV spectrophotometric method at 259 nm (EVO 300 PC, Thermo Nicolet, Thermo Fisher Scientific, Waltham, MA, USA) and supersaturation ratio (SR) was calculated as the ratio of the total compound concentration in the system to its equilibrium solubility.

In the second stage of solvent screening, preliminary LASP experiments were performed at S/AS values where highest SR was determined and additionally at SR values comparable between different solvents. In every case, both phases were passed through 0.22 or 0.45 µm nylon filter before application. An appropriate volume of cilostazol solution at a concentration close to saturation value in a given solvent was transferred into appropriate volume of water using a peristaltic pump (ISM404B V8.00, IsmaTec, Cole-Parmer GmbH, Wertheim, Germany) at 1 mL/min under magnetic stirring 250 rpm (MS-H-Pro+, Dragonlab, Beijing, China) at 25 °C; the total sample volume after mixing was kept constant at 20 mL (Figure 1B). Particle size distribution was determined immediately after precipitation as described in Section 3.6.1. 

### 3.3. Stabilizer Screening

To choose the most promising stabilizer (see Section 2.2) which allows to precipitate comparatively the smallest cilostazol particles, LASP screening studies were performed with the solvent and S/AS ratio chosen according to Section 2.1; the processing parameters were kept the same. The stabilizer concentration in water as antisolvent phase was set for screening purposes at 0.5% (*w*/*v*), except for SDS (0.002% to avoid exceeding critical micellar concentration and additional solubilization). Particle size distributions of the suspensions precipitated with different stabilizers were immediately measured to identify the smallest size. Additionally, selected stabilizer combinations were compared with the results of single excipient use. 

### 3.4. LASP–Study of Factors and Optimization Using DoE

The equipment used, sample volume (20 mL) and temperature (25°C) were kept the same as in the screening studies. Four independent variables at three levels were included (Table 5) in the face-centered central composite design (CCD). The plan consisted of 27 experimental points in total (Table 1) with three replicates of central point to estimate pure error in model fitting. Upper and lower levels of independent variables were selected to cover a relatively wide range while taking into consideration practical constraints. Maximum CIL concentration in the solvent phase was set at 100 mg/mL to avoid saturation of this phase. The weight ratio of stabilizer to cilostazol was selected based on the amounts typically reported for nanosuspensions (e.g., [67,87]), with the consideration that the excipient levels should be sufficiently low for the product to be regarded as nanocrystals. The mixing speed and peristaltic pump flow rate were chosen empirically based on the visual observation of effective stirring without foaming and differences in S dripping speed, respectively.

Linear and quadratic mathematical models, including interaction terms, were sought for the relationships between independent variables and four responses of interest describing PSD: d10, d50, d90 and span values. Statistically significant effects (*p* < 0.05) were identified with ANOVA and Pareto charts. The best refined model equation to characterize the influence of LASP parameters on particle size was chosen on the basis of the following statistical diagnostics criteria: (adjusted) coefficient of determination R^2^, R^2^adj., insignificant lack-of-fit test, normal distribution of residuals (i.e., the differences between the response values predicted by the model and observed values) according to Shapiro-Wilk test, as well as homoscedasticity (homogeneity of residuals).

For the selected optimal model, response surface maps (RSM) were constructed to graphically represent the relationships between LASP settings and response. Finally, based on the effects of independent variables and RSM shape, optimum values of the precipitation process parameters for the smallest achievable particle size were identified. Predicted responses were then calculated for these optimal settings. For model validation, experiments were carried out at the selected points, and prediction errors were calculated as %PE = (observed value − predicted value)/observed value.

All the calculations, including the generation of CCD and statistical model evaluation, were performed with STATISTICA 13.1 (StatSoft Polska, Cracow, Poland). 

### 3.5. Sonication–Study of Factors and Optimization

#### 3.5.1. Preliminary Sonication Studies

At this stage, the following parameters were kept constant: CIL concentration in the S phase (55 mg/mL), ultrasound (US) amplitude (60%) and sonication time of 25 min, to reflect the central values planned for the next stage of investigation (Section 3.5.2). S/AS ratio, PVA/CIL ratio of 1 and sample volume remained the same as in the previous development stage and sonication was performed with 6 mm probe connected to 130 W processor (VCX-130, Sonics & Materials Inc., Newtown, CT, USA) with the sample placed in ice bath throughout the process (Figure 1B).

One considered qualitative variable was the moment of ultrasound application, as in the context of nanosuspensions two variants are described: concomitant insonation during the solvent-antisolvent addition (sensu stricto sonoprecipitation), e.g., [67,88] and two-step LASP followed by immediate insonation (precipitation-sonication), e.g., [55,68]. Therefore, in a single-step process, the ultrasonic probe was activated immediately at the start of S phase addition. Due to technical constraints, no additional stirring was applied in this variant, since a stirrer bar would interfere with the probe or the probe could vibrate in air if stronger vortex formed during mixing. On the other hand, the two-step process started with LASP (with stirring at 600 rpm, S flow rate 3 mL/min) and immediately after the precipitation, ultrasound was applied. 

Another consideration was to compare the effect of different insonation modes, continuous vs. intermittent. Continuous sonication generally results in smaller particles [23], but simultaneously may overheat the sample, which may reduce supersaturation, decrease the nucleation rate or cause secondary dissolution of newly precipitated nanoparticles. Therefore, intermittent ultrasound was also tested, with 5 s pulses and 5 s intervals for 25 min in total. The comparison was made only for two-step LASP-sonication due to the difficulty in synchronizing S phase addition with pulsed insonation. 

To further elucidate the parameters potentially affecting CIL PSD, two temperatures of AS phase at the start of the process were compared, i.e., 25 °C vs. 11 °C. Although temperature may have a critical influence on precipitation outcome as one of the factors determining the supersaturation and nucleation rate, and in some cases cooling of the sample was reported as necessary to obtain nanosized particles [56], due to the equipment limitations it was not possible to set precise temperature in the wider range and control it throughout the whole process. For this reason, temperature was not included in quantitative DoE studies and instead was treated as a control variable. The preliminary study aimed to choose the starting AS temperature value to be kept during further experiments. 

#### 3.5.2. LASP+Sonication Study of Factors Using DoE

An orthogonal Central Composite Design was used for three independent variables at three main levels and axial points (Table 6). 

The CCD plan was executed in six replicates for central points and three replicates for other points. The responses of interest were the same PSD parameters as for the LASP characterization. Modeling and statistical evaluation was performed as described in Section 3.4. for precipitation process DoE.

### 3.6. Micro- and Nanocrystals Characterization Methods

#### 3.6.1. Particle Size Distribution (PSD)

Particle size distribution of every precipitated sample was immediately determined with laser diffraction technique (Mastersizer 3000 equipped with Hydro SV wet dispersion accessory, Malvern Panalytical Ltd., Malvern, UK). When measuring the PSD of nanosuspensions, care must be taken to avoid premature particle dissolution during the test on the one hand and additional precipitation upon diluting the sample with dispersant on the other hand [89,90]. To minimize the risk, the dispersant applied for every measured sample mimicked the composition of the precipitated (nano)suspension’s liquid phase, i.e., it consisted of appropriate proportion of solvent and antisolvent saturated with cilostazol. In the case of solvent screening, where stabilizer was absent from AS, 0.0125% HPMC was added to dispersant in order to improve particle wetting and prevent agglomeration during the measurement. Every dispersant was filtered through 0.22 µm nylon filters and its refractive index (RI) was measured (Optronic DR 201-95, A. Krüss Optronic GmbH, Hamburg, Germany) for proper calculation of diffraction data. After stabilizer screening, the dispersant RI was found to be fixed at 1.35.

Apart from dispersant RI, other measurement parameters were kept constant, as found during method development: background measurement time of 10 s, sample measurement time of 5 s, Hydro SV dispersion speed 1800 rpm, cilostazol RI = 1.57 and absorption index = 0.001 (same values for blue and red light reading). Samples appropriately diluted with dispersant were added to reach obscuration of ~5–15% in the case of microparticles or larger material and 2–7% in the case of nanoparticles. At least six measurements were recorded for every sample, and volume-based PSD values of d10, d50, d90 and span were determined. 

#### 3.6.2. Physical Stability and Process Efficiency

Physical stability of optimal suspensions’ size was evaluated by comparing the PSD determined for freshly precipitated samples and after 1, 3, 6, 24, 72 h, as well as 1 and 8 weeks of storage at 25 °C. 

The efficiency of both LASP and LASP+US processes was evaluated by measuring cilostazol concentration in the liquid phase remaining after precipitation. Depending on particle size, samples were filtered through 0.22 µm nylon (microsuspensions) or Anotop Plus 0.02 µm (nanosuspensions) syringe filters with the first few milliliters of the filtrate discarded. Dissolved cilostazol concentration was determined spectrophotometrically at 259 nm against the blank consisting of appropriate composition of solvent and antisolvent. Process efficiency related to the amount of suspended CIL was then calculated as: yield = (total mass of CIL in 20 mL sample introduced with S phase − mass of dissolved CIL in 20 mL sample)/total mass of CIL. 

#### 3.6.3. Solid State and Morphology Characterization

For solid state analyses nanosuspension samples were centrifuged at 10,000 rpm for 10 min and the supernatant was removed, while microsuspension samples were collected by vacuum filtration on 0.45 µm nylon filter. The samples were washed with stabilizer solution and oven dried at 50 °C, then the powder was submitted to standard tests to identify the material’s (pseudo)polymorphic form and any transitions with respect to untreated cilostazol.

DSC analyses were carried out using differential scanning calorimeter DSC8500 (Perkin Elmer, Waltham, MA, USA) in the temperature range 20–150 °C at heating rate 10 °C/min under nitrogen flow of 20 mL/min. TGA scan (TGA 4000, Perkin Elmer, Waltham, MA, USA) was applied for selected samples and employed the same heating and flow rates in temperature range 30–600 °C. 

XRPD was performed with powder diffractometer D8Advance (Bruker, Billerica, MA, USA) equipped with Johansson monochromator (λCu Kα1 = 15,406 Å) LynxEye strip detector. Samples were pressed into a cuvette and measurement was taken in 2θ angle range of 4–39, with step value of 0.025 and step time of 1.5 s. 

FTIR spectra were registered using Jasco 4700A (Jasco Inc., Easton, MD, USA) spectrometer in the 4000–400 cm^−1^ range in transmittance scale with 1 cm^−1^ resolution. Powder samples (1–2 mg) were mixed in mortar with KBr (250 mg) and compressed under 100 bar pressure, while kept under vacuum for deaeration. 

SEM imaging was performed with Quanta FEG 250 (FEI, Thermo Fisher Scientific, Waltham, MA, USA) microscope in low vacuum conditions under 70 Pa pressure and accelerating voltage of 10 kV. For AFM imaging, suspended samples were deposited on freshly cleaved mica and left to evaporate. The analysis was carried out in intermittent contact mode with AFM microscope Agilent 5500 (Agilent, Santa Clara, CA, USA), using All-In-One A1 probe (Budget Sensors, Sofia, Bulgaria) and cantilever C with force constant of 7.4 N/m and resonance frequency 150 kHz. For TEM imaging, suspended samples were transferred to a carbon-coated copper grid and stained with 2% phosphotungstic acid over 2 min. Images were collected using Hitachi HT7700 transmission electron microscope (Hitachi High-Tech, Tokyo, Japan) in high contrast mode at 100 kV voltage.

#### 3.6.4. Solubility Study and Dissolution Testing

Apparent or kinetic solubility [91,92] of cilostazol nanoparticles was compared with thermodynamic solubility of raw CIL to verify if an increase can be observed. Two media were considered: water and ~0.1% solution of PVA, i.e., the antisolvent phase, in order to separate any potential influence of polymer-cilostazol interaction on the drug’s saturation solubility and to stabilize possible supersaturation state resulting from the dissolution of nanocrystals. 

An excess of raw cilostazol or nanosuspension was added to the appropriate medium and shaken at 25 °C and 37 °C (n = 3). After 24 and 72 h the sample aliquots were filtered with 0.22 µm nylon syringe filters (raw CIL) or Anotop 0.02–0.1 µm syringe filters with the first few milliliters of the filtrate discarded and cilostazol concentration was measured spectrophotometrically to determine solubility value. 

The experimental apparent solubility values of nanocrystals were compared with the theoretical calculations of Ostwald-Freundlich equation (Equation (2)). Particle radii were derived from d10, d50, d90 values of the studied nanosuspension, cilostazol MW = 369.467 g/mol, ρ = 1.26 g/cm^3^, γ = 72 mN/m for water and 57 mN/m for AS phase were taken from the literature [14,93]. 

To confirm the effect of reduced PSD on cilostazol dissolution rate, tests were carried out using USP 2 apparatus (Erweka DT 126 Light, Erweka GmbH, Langen, Germany) with 500 mL water as dissolution medium at 37 °C and paddle speed of 25 rpm. (Nano)suspension amounts corresponding to 3 mg of cilostazol were introduced with a pipette to the bottom of dissolution vessels. Medium samples of 5 mL were withdrawn without replacement at 1, 3, 5, 10, 15, 20, 30 and 60 min and filtered as soon as possible through Anotop Plus 0.02 µm syringe filters (nanosuspensions) or nylon 0.22 µm syringe filters (microsuspension and coarse suspension). Dissolution profiles (% of the dose dissolved) were calculated based on the spectrophotometric measurement of cilostazol concentration in the samples, and similarity factor f2, and first order dissolution rate constant (k1) were evaluated with DDSolver [94] (n = 6).

## 4. Conclusions

Liquid antisolvent precipitation process combined with sonication was systematically studied with the aim to optimize the conditions for particle size reduction of cilostazol, a BCS class II drug. Initial screening studies were carried out to compare and choose an appropriate solvent phase, determine its proportion to water as antisolvent, and to identify the most useful stabilizer. Experiments performed according to Central Composite Design matrices revealed significant influence of such precipitation factors as feed concentration, stabilizer amount and stirring speed, while for LASP combined with sonication, feed concentration and ultrasonic energy (time and amplitude) were found to be critical. The results served to optimize processing conditions for minimal achievable particle size. 

Optimized liquid antisolvent precipitation alone was unable to produce cilostazol nanosuspension, but was effective in size reduction to microcrystals (d10 = 2.70, d50 = 4.93, d90 = 8.39 µm). Applying sonication immediately after LASP was necessary to obtain nanosized material. As a result of systematic process characterization and optimization, stable cilostazol nanosuspensions (d10 = 0.06, d50 = 0.33, d90 = 1.45 µm) were produced with the following settings: 10% DMSO as solvent phase, cilostazol feed concentration 10 mg/mL, PVA/CIL weight ratio 1.0, stirring during precipitation 600 rpm, sonication amplitude 100%, sonication time 60 min. Both precipitation and its combination with ultrasound did not change cilostazol’s original crystalline form A. Nanosuspension displayed higher apparent solubility than the compound’s equilibrium solubility value, and the dissolution rate was improved not only over coarse material, but also in comparison to microsuspension. Therefore, the study demonstrated that, with systematic evaluation of processing variables and optimization, a bottom-up method is successful in nanosizing of cilostazol for the dissolution improvement dissolution of this poorly water soluble, BCS class II drug. 

## Figures and Tables

**Table 1:** Independent variable settings and results of central composite design experiments used in liquid antisolvent precipitation optimization and characterization. ‘(C)’ marks central points of the plan.

Run No. (Randomized Order)	Independent Variables Settings				Dependent Variables Results			
	CIL Conc. [mg/mL]	PVA/CIL	Solvent Phase Flow Rate [mL/min]	Mixing [rpm]	d10 [µm]	d50 [µm]	d90 [µm]	Span
23	55	0.75	3	250	3.22	5.74	9.84	1.153
22	55	0.75	5	500	3.07	5.59	9.56	1.161
21	55	0.75	1	500	2.42	5.31	10.30	1.482
25 (C)	55	0.75	3	500	2.81	4.82	7.60	0.994
27 (C)	55	0.75	3	500	2.32	4.27	7.38	1.183
5	10	1.25	1	250	2.39	5.40	12.10	1.806
13	100	1.25	1	250	3.16	5.48	9.03	1.069
1	10	0.25	1	250	5.22	15.00	28.60	1.563
4	10	0.25	5	750	4.47	9.49	17.40	1.368
26 (C)	55	0.75	3	500	3.20	5.32	8.51	0.999
15	100	1.25	5	250	2.91	5.52	9.79	1.247
24	55	0.75	3	750	2.38	4.05	6.39	0.991
9	100	0.25	1	250	4.88	10.80	20.40	1.433
10	100	0.25	1	750	4.43	9.53	18.10	1.439
3	10	0.25	5	250	6.54	23.00	41.50	1.518
12	100	0.25	5	750	5.77	11.40	19.60	1.220
14	100	1.25	1	750	3.02	5.15	8.28	1.021
11	100	0.25	5	250	5.35	11.20	19.30	1.253
8	10	1.25	5	750	2.75	4.97	8.40	1.136
20	55	1.25	3	500	2.79	4.89	8.01	1.066
7	10	1.25	5	250	3.28	6.39	12.10	1.384
16	100	1.25	5	750	2.82	5.30	9.04	1.174
18	100	0.75	3	500	2.81	5.11	8.58	1.130
17	10	0.75	3	500	4.03	8.69	17.30	1.526
2	10	0.25	1	750	4.93	12.70	23.80	1.486
19	55	0.25	3	500	8.67	16.80	28.70	1.194
6	10	1.25	1	750	2.06	3.45	5.63	1.032

**Table 2:** Summary of variable combinations and results (mean ± SD) of preliminary sonication studies (n = 3).

Setup No.	Moment of Ultrasound Application	Sonication Pattern	Initial AS Temperature [°C]	PSD [µm]		
				d10	d50	d90
1	single-step sonoprecipitation	continuous	11	2.74 ± 0.53	4.81 ± 1.14	8.21 ± 2.48
2	single-step sonoprecipitation	continuous	25	2.79 ± 0.02	5.09 ± 0.31	9.04 ± 0.88
3 n = 2	two-step LASP+sonication	continuous	11	2.54 ± 0.63	3.84 ± 0.63	5.73 ± 0.33
4	two-step LASP+sonication	continuous	25	2.10 ± 0.06	3.70 ± 0.10	6.25 ± 0.13
5	two-step LASP+sonication	pulse 5 s + pause 5 s	11	5.26 ± 0.23	8.58 ± 0.38	13.20 ± 0.70
6	two-step LASP+sonication	pulse 5 s + pause 5 s	25	2.16 ± 0.20	3.96 ± 0.43	6.80 ± 0.64

**Table 3:** Independent variable settings and results of central composite design experiments used in LASP+sonication optimization and characterization.

Run No. (Randomized Order)		Independent Variables Settings				Dependent Variables			
	Rep.	CIL Conc. [mg/mL]	Amplitude [%]	Time [min]	^a^ Energy [J]	d10 [µm]	d50 [µm]	d90 [µm]	Span
39	3	90	90	10	13,808	2.220	3.950	6.60	1.11
17	2	20	30	10	2994	2.070	3.790	6.67	1.21
5	1	90	30	10	2995	2.520	4.550	7.54	1.12
26	2	100	60	25	19,394	2.350	4.210	6.96	1.09
33	3	20	30	10	2981	2.330	4.480	7.98	1.26
21	2	90	30	10	2994	2.410	4.230	6.94	1.07
12	1	55	99	25	40,472	1.840	3.170	5.47	1.15
24	2	90	90	40	n/a	2.120	3.800	6.48	1.15
14	1	55	60	44	31,862	1.940	3.370	5.74	1.13
7	1	90	90	10	11,802	2.460	4.750	8.12	1.19
3	1	20	90	10	13,254	1.830	3.180	5.49	1.15
28	2	55	99	25	39,596	1.830	3.180	5.49	1.51
15 (C)	1	55	60	25	19,497	2.050	3.630	6.20	1.14
47 (C)	3	55	60	25	19,494	2.080	3.650	6.15	1.12
30	2	55	60	44	33,469	1.970	3.530	6.10	1.17
27	2	55	21	25	4504	2.490	4.570	7.65	1.13
* 20	2	20	90	40	54,540	* 0.032	* 0.306	* 4.43	14.36
29	2	55	60	6	4318	2.220	3.850	6.33	1.10
* 36	3	20	90	40	54,438	* 0.031	* 0.314	* 3.91	12.37
45	3	55	60	6	4312	2.200	3.830	6.32	1.08
31 (C)	2	55	60	25	19,495	2.160	3.820	6.39	1.11
* 41	3	10	60	25	17,993	* 0.029	* 0.233	* 4.09	17.45
34	3	20	30	40	12,009	1.970	3.440	5.90	1.14
48 (C)	3	55	60	25	18,002	2.030	3.700	6.44	1.19
37	3	90	30	10	2995	2.640	4.820	8.18	1.15
* 9	1	10	60	25	18,008	* 0.029	* 0.245	* 4.62	18.81
18	2	20	30	40	12,007	2.280	4.090	6.81	1.11
32 (C)	2	55	60	25	18,005	2.170	3.800	6.32	1.09
43	3	55	21	25	4503	2.120	3.630	6.00	1.07
* 25	2	10	60	25	18,010	* 0.028	* 0.206	* 4.26	20.56
16 (C)	1	55	60	25	17,998	2.190	3.870	6.43	1.10
38	3	90	30	40	12,006	2.200	3.950	6.66	1.13
22	2	90	30	40	11,997	2.290	4.150	7.01	1.14
40	3	90	90	40	54,223	2.150	3.790	6.39	1.12
13	1	55	60	6	4317	2.240	3.970	6.60	1.10
11	1	55	21	25	4487	2.440	4.330	7.12	1.08
42	3	100	60	25	18,014	2.380	4.120	6.61	1.03
23	2	90	90	10	13,215	2.170	4.050	7.10	1.22
2	1	20	30	40	11,998	1.800	3.550	6.56	1.34
10	1	100	60	25	18,002	2.780	5.260	8.89	1.16
8	1	90	90	40	54,578	2.070	3.610	6.08	1.11
19	2	20	90	10	13,546	1.900	3.250	5.52	1.11
46	3	55	60	44	31,717	1.950	3.450	5.96	1.16
35	3	20	90	10	13,216	2.090	3.490	5.67	1.03
1	1	20	30	10	2999	2.090	3.620	6.07	1.10
* 4	1	20	90	40	53,803	* 0.033	* 0.385	* 4.21	10.87
44	3	55	99	25	39,061	2.040	3.620	6.23	1.16
6	1	90	30	40	12,007	2.250	3.930	6.44	1.07

(C) marks central points of the plan. ^a^ Recorded results to be treated as additional variable as a function of time and amplitude. * denotes results with nanoparticulate fractions.

**Table 4:** Settings and results (mean ± SD) of the optimization study for sonoprecipitation (n = 3).

Setup No.	CIL Concentration [mg/mL]	Moment of Ultrasound Application	Initial AS Temperature [°C]	PSD [µm]		
				d10	d50	d90
1	10	single-step sonoprecipitation	25	0.030 ± 0.004	0.191 ± 0.05	3.49 ± 0.21
2	10	single-step sonoprecipitation	11	0.030 ± 0.004	0.181 ± 0.05	3.48 ± 0.39
3	10	two-step LASP+sonication	11	0.028 ± 0.01	0.162 ± 0.02	3.09 ± 0.56
4	10	two-step LASP+sonication	25	0.045 ± 0.05	0.582 ± 0.15	3.71 ± 0.29
5	20	single-step sonoprecipitation	11	1.28 ± 1.06	2.85 ± 0.48	5.04 ± 0.16
6	20	two-step LASP+sonication	11	0.74 ± 1.12	2.82 ± 0.46	5.16 ± 0.32

**Table 5:** Independent variables and their levels used in Central Composite Design studies for LASP characterization and optimization.

Independent Variable	Level		
	−1	0	+1
CIL concentration in solvent phase [mg/mL]	10	55	100
PVA/CIL ratio [*w*/*w*]	0.25	0.75	1.25
Solvent phase flow rate [mL/min]	1	3	5
Mixing speed [rpm]	250	500	750

**Table 6:** Independent variables and their levels used for Central Composite Design studies for LASP+sonication characterization and optimization.

Independent Variable	Level				
	−α	−1	0	+1	+α
CIL concentration in S phase [mg/mL]	10	20	55	90	100
Ultrasound amplitude [%]	21	30	60	90	99
Sonication time [min]	6	10	25	40	44

**Figure A1:**
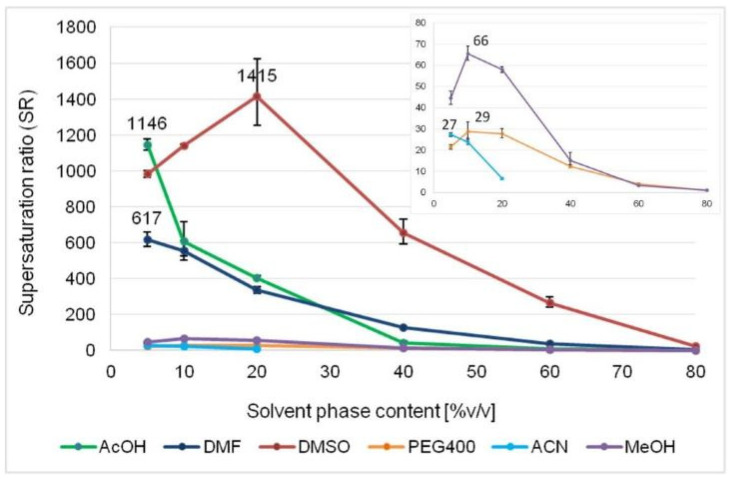
Cilostazol supersaturation ratios at different compositions of organic solvents and water.

**Figure A5:**
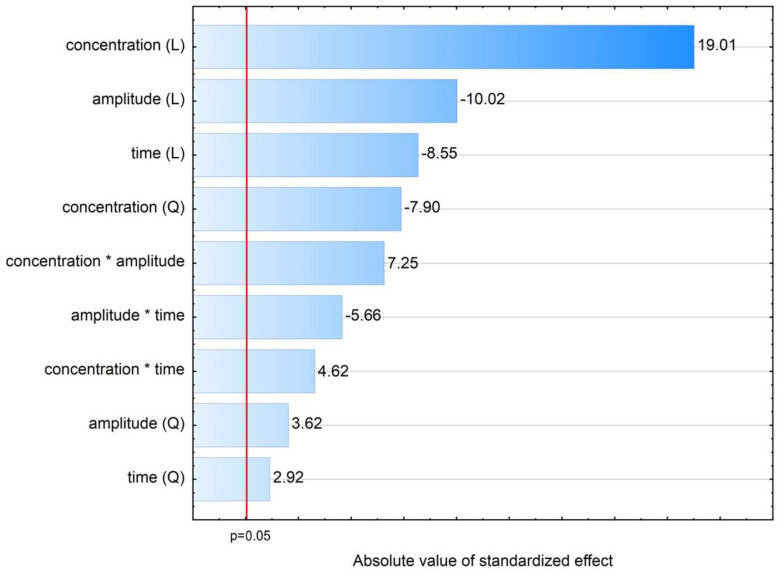
Pareto chart of the standardized linear (L) and quadratic (Q) effect sizes of independent variables and their interactions in precipitation+sonication process studied according to central composite design plan. Bars above the red line denote statistically significant effects at *p* < 0.05.

## Data Availability

Data is contained within the article.

## Appendix A. Details of Solvent and Stabilizer Screening

Cilostazol solubility in different solvents was determined as a part of solvent screening stage (Section 2.1). As expected, cilostazol equilibrium solubility increased with increasing organic solvent content with the exception of acetonitrile, where solubilities were higher at 60% and 80% contents of organic phase than in the 100% solvent–therefore, for ACN the supersaturation ratios were evaluated up to 20%. Among the studied options, the highest solubility in pure solvent was recorded for acetic acid (223±11 mg/mL) and the lowest for PEG400 (3.8 ± 0.4 mg/mL), which is consistent with the literature [30]. The highest SR value, expected to be most favorable for fast nucleation, was different for different solvents (Figure A1). Based on these values the following S/AS combinations were chosen for screening LASP: 5% AcOH, 5% DMF, 20% DMSO, 5% ACN, 10% MeOH and 10% PEG. To further explore different solvent properties, additional points were examined: 10% DMSO (SR of 1139 comparable to that of 5% AcOH), 10% AcOH and 40% DMSO (SR of 605 and 665 comparable to that of 5% DMF).

Regarding the size of the particles precipitated at the abovementioned SR conditions, with the exception of ACN and PEG400 samples, particle size distributions were broad or polymodal. Interestingly, the results of LASP from acetic acid turned out to be the poorest, especially when compared to DMF and DMSO at similar supersaturation ratios (Figure A2).

In the solvent screening studies, a qualitative correlation was detected between solvent polarity and the precipitated particles’ size (Table A1). These results are in general agreement with the observations made by Chung et al., who note that for poorly water soluble compounds in polar solvents, molecular clusters of the precipitated compound form rapidly and nucleation is immediate due to fast mutual diffusion of solvent and water. On the other hand, for substances of relatively higher aqueous solubility the opposite was observed, i.e., their particle size increased with increasing solvent polarity [31]. On the contrary, the reverse interpretation of drug solubility-solvent polarity interplay has been made by Beck et al., who concluded that poorly water-soluble APIs precipitate as smaller particles from nonpolar solvents, while better soluble ones–from more hydrophilic solvents. It must be noted however that Beck et al.’s study was done in a system containing PEG as stabilizer in antisolvent phase instead of pure water, which affected the proposed mechanism. According to these authors, poorly soluble API molecules diffuse slowly in less polar solvents and vice versa, and consequently particle growth on the interfacial surface is slower, allowing for stabilizer to adsorb more effectively [95]. Stabilizer was also present in a study where DMSO produced smaller griseofulvin particles than acetone and ethanol, which was attributed to its higher density, polarity and viscosity [96].

Considering this, direct comparisons between solvent screening results in the present study and similar papers lack common ground. The current results cannot easily be referred to other precipitation conditions or drugs either, as according to some reports, no universal correlations between solvent properties and precipitated particles’ size can be found [97,98].

**Table A1:** Selected properties of the screened solvents (based on PubChem data or supplier data sheets unless otherwise noted).

Solvent	MW	Dielectric Constant	Viscosity [mPas]	Density [g/cm^3^]	Log P	Surface Tension [mN/m]
DMSO	78.14	47 ^a^ [99]	2.47 ^b^	1.1	−1.35	42.27 ^c^ [100]
DMF	73.09	38 ^a^ [99]	0.802 ^a^	0.95	−1.01	36.42 ^a^
MeOH	32.00	33 ^b^ [101]	0.544 ^a^	0.79	−0.77	22.07 ^a^
PEG 400	400	12.50 ^a^ [102]	120 ^b^	1.13	n/a	42.06 ^c^ [103]
ACN	41.05	38.8 ^b^	0.35 ^b^	0.79	−0.34	29.04 ^b^
AcOH	60.05	6.15 ^a^ [104]	1.056 ^a^	1.05	−0.17	27.10 ^a^

^a^ 25 °C, ^b^ 20 °C, ^c^ 30 °C.

During the stabilizer screening studies in the next step, for the same chemical type of polymers, a trend was observed between polymer MW or viscosity and the precipitated CIL PSD. HPMC grades generating lower viscosities (E6: 6 cP < E50: 50 cP < K4M: 4000 cP in 2% solution, according to product information), as well as smaller molecular weights of PVA (9–10 kDa: 3 cP < 30–70 kDa: 5 cP in 4% solution according to product information) resulted in smaller cilostazol particles (Figure 2 and Figure A3). This is most likely due to their increased diffusivity and facilitated adsorption of comparatively shorter polymeric chains on cilostazol crystal surfaces hindering excess growth. On the other hand, the opposite was observed for nonionic surfactant Poloxamer (PX188: 7680–9510 g/mol > PX407: 9840–14,600 g/mol;), which might possibly be caused by stronger hydrophobic interaction between cilostazol and polypropylene oxide block in PX407. Moreover, PX407 exhibits lower HLB values and surface tension in comparison to PX188, which contributes to higher nucleation rates. Interestingly, when compared to precipitation from 10% DMSO without stabilizers, PSD curves generally displayed single population of particles, and apart from 0.002% SDS no nanoparticulate fractions were detected (Figure A2 and Figure A3). This may suggest that nucleation conditions are more uniform than in pure S/AS or, more likely, nucleation rate was delayed in the presence of polymers [80,105].

For stabilizer combination screening studies, the concentration of SDS in the AS phase was increased to 0.1%. Although 0.002% employed previously is a value below critical micellar concentration of this surfactant, it appeared too low to significantly affect the LASP process, as revealed by the general similarity of its PSD curve with separate nanoparticulate fraction to the curve observed without any stabilizers (Figure A2 and Figure A3). Also for comparison purposes DOSS concentration was lowered to 0.1%, because the value of 0.5% seemed to cause secondary solubilization and redissolution upon the precipitation of cilostazol introduced with the first few drops of solvent phase.

There have been various attempts to correlate nanosuspension particle size to stabilizer properties, mostly in the framework of wet milling process, such as surface energy values similar to the drug’s, wettability [106,107], or molecular weight and polymer chain length [108]. In the case of bottom-up processes, a correlation has been found between stabilizer HLB value and the precipitated particle sizes, and explained by the similarity of LASP mechanism to emulsification, i.e., more lipophilic stabilizers are thought to interact stronger with hydrophobic APIs [34]. However, up to date universal relationships between polymer/surfactant properties and particle size have not been established, as the findings are often contradictory. Therefore, the choice of the optimal stabilizer for a particular drug processed with a particular method remains empirical and relies on trial and error method, guided by particle size results [33,108]. This is due to the complexity of API-stabilizer interaction mechanisms, where polymer/surfactant properties such as surface energy, chain structure, hydrophobicity or functional group interactions all play a role [33].

## Appendix B. Details of Statistical Analyses in DoE Studies on LASP and LASP+Sonication

The correlation between the observed ln d90 values and those predicted by the final model developed for LASP process (Section 2.3) is presented in Figure A4.

For the identification of target LASP conditions and simultaneous statistical model validation as described by Equation (3), preliminary optimal range was chosen based on the RSM and point predictions of ln d90 values were made for settings combination within this range, with further retransformation of the results to raw d90 values. According to the model, the smallest particles of d90 = 5.56 µm would be produced at 50 mg/mL CIL in the solvent phase, PVA/CIL ratio of 1 and under 750 rpm stirring. These settings were chosen for model validation experiments (n = 6). For comparison purposes and further evaluation of the model’s predictive utility, another set of validation experiments (n = 4) was also carried out with LASP parameters set arbitrarily at the values closer to the center of RSM design space: 20 mg/mL CIL in the solvent phase, PVA/CIL ratio of 1 and 620 rpm stirring. Since the effect of flow rate was statistically insignificant, it was kept at 5 mL/min.

The results of LASP validation experiments are given in Table A2. It is worth noting that the model’s prediction intervals are broad. On the one hand, this may be an effect of building the model equation on ln-transformed response values and the fact that retransformed values with their units in µm are more sensitive to any prediction uncertainties. On the other hand, this may also be explained by the fact that R^2^ = 0.91 (R^2^adj. = 0.88), while reasonable and not uncommon in DoE reports [44,57,109,110,111], means that 12% of response variability remains unpredicted and not accounted for by the model. This is most likely related to uncontrolled noise and processing fluctuations, for instance such as slight hydrodynamic changes induced in the system by minor shifts in vessel or nozzle positioning, or random external impurities modifying nucleation uniformity.

**Table A2:** Results of validation experiments for the model describing the relationship between LASP process variables and ln d90 value.

Settings	Predicted ln d90 (95% Prediction Interval)	Predicted d90 [µm] (95% Prediction Interval)	Observed d90 [µm] (Min–Max)	Prediction Error (PE) [%]
50 mg/mL CIL PVA/CIL = 1.0 750 rpm	1.72 (1.33–2.10)	5.56 (3.79–8.13)	8.74 (7.71–10.8)	36
20 mg/mL CIL PVA/CIL = 1.0 620 rpm	2.13 (1.78–2.48)	8.44 (5.94–11.97)	8.39 (7.48–9.13)	0.6

Moreover, as seen in Table A2, the validation experiment under the identified optimal conditions resulted in a very large prediction error (PE) of 36% and the observed mean value of 8.74 µm considerably exceeded the calculated value. This relatively poor predictive power might be explained by the fact that the experiment was carried out at the +1 level of stirring speed, i.e., on the edge of the experimental space, where PE is usually higher than closer to its center. Accordingly, prediction was markedly (60 times) improved for the second set of validation experiments (PE = 0.6%). Besides this, single exploratory attempts were made at precipitation at the highest possible stirring speed of 1500 rpm (above the range covered by DoE), however without any major improvement in particle size reduction (d90~7–8 µm).

Regarding the analysis of LASP+sonication DoE results (Table 3), first attempt at modeling the relationship between independent variables and d50 with linear, quadratic and factor interactions effects revealed that all the effects were statistically significant (Figure A5). A model equation which would include all these terms suffered from poor diagnostic criteria i.e., low regression coefficients (R^2^ = 0.80, R^2^adj. = 0.75) and significant lack-of-fit statistics. Any attempts to reduce the number of model parameters to incorporate only the strongest effects or mathematically transform the dependent variable did not improve the diagnostics.

Therefore, a different approach was taken to find a simplified model with acceptable diagnostics and fewer terms to avoid overparametrization and facilitate interpretation. Specifically, since both sonication time and amplitude exerted linear, quadratic and interaction effects, these two independent variables were substituted with a new single variable: ultrasound energy supplied to the system. While not a process setting that can be controlled directly, it was a value recorded for the processing of every sample (Table 3), correlated positively to both sonication amplitude and time (R^2^ = 0.997), where longer insonation times are able to compensate low amplitudes and vice versa to a certain extent.

Initially, a full model including linear, quadratic and interaction effects of CIL concentration and energy was explored, but it could not be employed due to low R^2^= 0.79 and unfulfilled assumption of normal distribution of residuals. Residual analysis revealed outlier values for the results of runs 41, 9 and 25, i.e., the axial points where feed concentration was set at 10 mg/mL (Table 3, Figure A6A). Thus, these central composite design plan points were deliberately excluded from further analysis. This exclusion markedly improved regression coefficients (R^2^ = 0.91, R^2^adj. = 0.89) and served as the basis for further refinement, as normality of residuals still was not satisfied. Exclusion of insignificant terms yielded the final model for d50 (Equation (4)) with acceptable distribution of residuals (Figure A6B).
